# SIRT6 transcriptionally regulates global protein synthesis through transcription factor Sp1 independent of its deacetylase activity

**DOI:** 10.1093/nar/gkz648

**Published:** 2019-08-02

**Authors:** Venkatraman Ravi, Aditi Jain, Danish Khan, Faiz Ahamed, Sneha Mishra, Malyasree Giri, Meena Inbaraj, Swati Krishna, Mohsen Sarikhani, Sangeeta Maity, Shweta Kumar, Riyaz Ahmad Shah, Pratik Dave, Anwit S Pandit, Rajprabu Rajendran, Perumal A Desingu, Umesh Varshney, Saumitra Das, Ullas Kolthur-Seetharam, Sona Rajakumari, Mahavir Singh, Nagalingam R Sundaresan

**Affiliations:** 1 Department of Microbiology and Cell Biology, Indian Institute of Science, Bengaluru, India; 2 Centre for BioSystems Science and Engineering, Indian Institute of Science, Bengaluru, India; 3 Molecular Biophysics Unit, Indian Institute of Science, Bengaluru, India; 4 Department of Molecular Reproduction, Development and Genetics, Indian Institute of Science, Bengaluru, India; 5 Tata Institute of Fundamental Research, Colaba, Mumbai, India; 6 NMR Research Centre, Indian Institute of Science, Bengaluru, India

## Abstract

Global protein synthesis is emerging as an important player in the context of aging and age-related diseases. However, the intricate molecular networks that regulate protein synthesis are poorly understood. Here, we report that SIRT6, a nuclear-localized histone deacetylase represses global protein synthesis by transcriptionally regulating mTOR signalling via the transcription factor Sp1, independent of its deacetylase activity. Our results suggest that SIRT6 deficiency increases protein synthesis in mice. Further, multiple lines of *in vitro* evidence suggest that SIRT6 negatively regulates protein synthesis in a cell-autonomous fashion and independent of its catalytic activity. Mechanistically, SIRT6 binds to the zinc finger DNA binding domain of Sp1 and represses its activity. SIRT6 deficiency increased the occupancy of Sp1 at key mTOR signalling gene promoters resulting in enhanced expression of these genes and activation of the mTOR signalling pathway. Interestingly, inhibition of either mTOR or Sp1 abrogated the increased protein synthesis observed under SIRT6 deficient conditions. Moreover, pharmacological inhibition of mTOR restored cardiac function in muscle-specific SIRT6 knockout mice, which spontaneously develop cardiac hypertrophy. Overall, these findings have unravelled a new layer of regulation of global protein synthesis by SIRT6, which can be potentially targeted to combat aging-associated diseases like cardiac hypertrophy.

## INTRODUCTION

Living cells are constantly engaged in the process of synthesizing and degrading proteins in a highly organized manner. Under physiological conditions, protein synthesis warrants a significant investment of cellular energy resources, often competing with mechanisms of cellular repair and maintenance. The protein synthesis thus is a tightly regulated process and dysregulation of proteostatic mechanisms negatively impacts the overall health of the cell. Protein synthesis happens to be one of the basic downstream cellular processes targeted by signalling pathways implicated in aging (1). Importantly, down-regulation of protein synthesis improves longevity in model organisms (2). Reducing protein synthesis has been shown to lower the accumulation of misfolded, aggregated or damaged proteins (3). However, the intricate signalling pathways that link protein synthesis with aging are not well understood.

Sirtuins are a class of highly conserved NAD^+^ dependent deacetylases best noted for their role in aging and aging-associated pathologies (4). The founding member of this family is the yeast Sir2 (silencing information regulator 2) and the homologs of Sir2 have been shown to extend lifespan in lower organisms including yeast, worms, and flies (5–7). Seven mammalian homologs SIRT(1–7) have been described, which have distinct sub-cellular localization and regulate diverse cellular functions including energy metabolism, cellular stress resistance, genomic stability, aging, and tumorigenesis (8). While SIRT1 and SIRT2 are localized both in the nucleus and cytoplasm, SIRT3, SIRT4 and SIRT5 are predominantly localized in mitochondria. SIRT7 is found in the nucleolus (8,9). Sirtuin 6 (SIRT6), is a chromatin-associated, nuclear-localized sirtuin, best characterized for its NAD^+^-dependent deacetylation of histone lysine residues H3K9 and H3K56 (10). It affects a broad range of cellular functions such as metabolism, DNA repair, inflammation, telomere maintenance, and is a key player in heart disease, cancer, diabetes, obesity and aging (10). SIRT6 knockout mice suffer from severe hypoglycaemia, loss of subcutaneous fat, a curved spine and lymphopenia resembling a progeroid like syndrome. They develop normally until 2 weeks after birth but undergo accelerated aging and die within 1 month of age (11). The cellular events that contribute to the aging and the associated complications under SIRT6 deficiency are just beginning to be understood.

One of the master regulators of protein synthesis inside the cell is the nutrient and energy sensor kinase mechanistic target of rapamycin (mTOR). mTOR is a serine/ threonine protein kinase that belongs to the family of phosphoinositide 3-kinase (PI3K)-related kinase. mTOR protein organizes itself into two multiprotein complexes mTORC1 and mTORC2 each with distinct subunit composition and functions, of which the mTORC1 is involved in the regulation of protein synthesis (12). The mTORC1 integrates signals from multiple extracellular and intracellular cues to regulate a battery of catabolic and anabolic processes including protein synthesis, autophagy, lipid synthesis and energy metabolism (13). In the presence of growth stimulatory signals, Rheb, an upstream GTPase, recruits mTORC1 to the surface of lysosomes and stimulates the kinase activity of mTOR. Activation of mTORC1 leads to phosphorylation of its downstream targets p70S6K and 4EBP1, which directly leads to an increase in the overall protein synthesis (13,14).

In the present study, we find that SIRT6 acts as a key regulator of cellular protein synthesis by transcriptionally regulating the mTOR signalling in partnership with the transcription factor Sp1.

## MATERIALS AND METHODS

### Cell culture, transfection and generation of stable cell lines

Cells were grown in high glucose DMEM supplemented with 10% fetal bovine serum and antibiotic-antimycotic mix. Cells were maintained at 37°C and 5% CO_2_ in a humidified incubator. For transfection, cells were grown till 70–80% confluence and the plasmids were transfected using Lipofectamine® 2000 reagent according to the manufacturer's protocol. Lipofectamine RNAiMAX was used for transfection of siRNAs. For generation of SIRT6 stable knockdown cell lines, HEK 293T cells were transfected with pAmpho Retro and the pSUPERretro-Sirt6 shRNA1 or control plasmid. 48 h post transfection the viral particles harbouring the shRNA were collected from the culture supernatant and three rounds of infection were carried out on a fresh stock of cells followed by 2 weeks of puromycin selection. For generation of stable Sp1 knockout cell line, gRNA targeting Sp1 cloned in plentiCRISPR v2 vector was obtained from GenScript^®^. Control vector (plentiCRISPR v2) or the Sp1 gRNA construct were co-transfected with lentiviral packaging constructs psPAX2 and pMD2.G in HEK 293T cell line. The resulting viral particles were then used to infect the HeLa cells and was followed by puromycin selection to generate stable knockout cell lines. For adenovirus transduction, the cells were infected with equal MOI’s of SIRT6 or null adenoviral preparations amplified in HEK 293T cell line. Primary culture was performed as described previously (15 ).

### Generation of SIRT6 mutants

Catalytic mutants SIRT6-H133Y and SIRT6-S56Y were generated by site-directed mutagenesis of the wild-type SIRT6-Flag plasmid using the QuikChange Site-Directed Mutagenesis Kit. The mutation was confirmed by sequencing.

### Animal studies

All animal studies were performed after obtaining due approval from the Institutional animal ethics committee of Indian Institute of Science, Bengaluru, India. The procedures were carried out in strict accordance with the recommendations of the Committee for the Purpose of Control and Supervision of Experiments on Animals (CPCSEA), Government of India. The animals were fed a normal chow diet and maintained in well-ventilated cages under a 12 h light/dark cycle. For studies on mTOR signalling, age-matched wild-type and SIRT6 knockout mice were sacrificed at ∼24–26 days of age. For studies on protein synthesis by SUnSET assay, 2–3 months old age-matched wild-type and SIRT6 heterozygous mice were used. For the generation of muscle-specific SIRT6 knockout mice (mSIRT6-KO), homozygous SIRT6 floxed mice were bred with ACTA-Cre mice, which expresses Cre recombinase under the control of the alpha-skeletal actin (ACTA) promoter. Homozygous SIRT6 floxed mice were used as controls. For studies on mTOR signalling and the SUnSET analysis, 4–5 months old age-matched mSIRT6-KO and SIRT6 floxed mice were used. Cardiac function was assessed in 9 months old mice using Visual Sonics high-frequency ultrasound system as described previously (16). For rescue of cardiac hypertrophy, age-matched SIRT6 floxed controls and mSIRT6-KO mice (∼9-month-old) were injected intraperitoneally with vehicle or Torin1 at a dose of 20 mg/kg per day for 10 days.

### Preparation of cell lysates

Cells were harvested from culture dishes using a cell scraper and lysed with ice-cold cell lysis buffer (20 mM Tris–HCl pH 7.5, 150 mM NaCl, 1 mM EDTA, 1 mM EGTA, 1% Triton, 2.5 mM sodium pyrophosphate, 1 mM sodium orthovanadate, 1 mM PMSF and 1X protease inhibitor cocktail). For *in vivo* samples the tissues were lysed in RIPA buffer (20 mM Tris–HCl pH 7.5, 150 mM NaCl, 1 mM EDTA, 1 mM EGTA, 1% NP-40, 1% sodium deoxycholate, 2.5 mM sodium pyrophosphate, 1 mM sodium orthovanadate, 1 mM PMSF and 1X protease inhibitor cocktail). The lysates were cleared by centrifugation at 12 000 rpm for 10 min at 4°C and the supernatant was collected in a fresh tube.

### Electrophoresis and immunoblotting

Protein lysates were quantified using the Bradford assay and were normalized for an equal amount of protein. The lysates were then mixed with 2X Laemmli Sample Buffer with 5% β-mercaptoethanol, boiled at 96°C for 5 min and electrophoresed on 10% or 15% SDS-PAGE gels. The proteins were then transferred onto 0.45 μm PVDF membranes by overnight wet transfer. The membrane was blocked for 1 h with 5% milk prepared in TBST (Tris-buffered saline supplemented with 0.05% Tween 20) at room temperature followed by incubation at 4°C overnight with specific primary antibody prepared in 5% BSA in TBST (dilution range: 1:500 to 1:2500). Bound antibodies on the membrane were recognized by horseradish peroxidase-conjugated secondary antibodies incubated for 1 h at room temperature. Between each step, the blots were washed thrice for three minutes each, with TBST. Chemiluminescence was detected using ECL reagent and the images were acquired using a chemiluminescence imager.

### Immunoprecipitation assays

Cells were lysed in cell lysis buffer and 2 μg of specific antibody or normal rabbit IgG was added to 500 μg of total protein. The mixture was incubated overnight with rotation at 5 rpm in a rotator. The immunoprecipitated complexes were then captured on protein A sepharose beads, eluted in 2X Laemmli buffer and proteins were detected by western blotting. For assessing the effect of SIRT6 on deacetylation of Sp1, cells at ∼70% confluence were transfected with either wild-type SIRT6-WT or the SIRT6 mutant plasmids. Twenty four hours post transfection, the cells were washed with ice-cold 1X PBS and lysed in an ice-cold lysis buffer [50 mM Tris–Cl, pH 7.4, 150 mM NaCl, 1 mM EDTA, 1% Triton X-100, 5 mM NAM, 2 μM TSA and 1X protease inhibitor cocktail]. The lysates were cleared by centrifugation, and 500 μg of total protein from each lysate was incubated with Sp1 specific antibody. Lysates incubated with normal rabbit IgG was used as control. The immunoprecipitated complexes were then captured on protein A sepharose beads, eluted in 2X Laemmli buffer and the acetylation level of Sp1 was analysed by western blotting.

### SUnSET assay

*In vitro* and *in vivo* SUnSET assays were performed according to previously described protocols (17,18). Briefly, the cells were grown in cell culture dishes to 70–80% confluence. After the indicated treatments, the newly synthesized proteins were labelled by treating the cells with 1 μM puromycin for 30 min prior to harvesting the cells. The cells were then lysed and subjected to western blotting analysis. The puromycin labelled peptides were then detected using a specific anti-Puromycin antibody. For *in vivo* measurement of protein synthesis, the mice were injected with puromycin intraperitoneally at a dose of 40 nmol/g of body weight. The mice were sacrificed 30 min post puromycin administration and the tissues were harvested, snap frozen and stored in –80°C freezer, until further use. The lysates were prepared as described before and puromycin incorporation was analysed by western blotting.

### Polysome profiling

The procedure was carried out as described in a previous report with suitable modifications (19). Briefly, control and SIRT6 stable knockdown HEK 293T cells were grown in 150 mm dishes until 80–90% confluence. Prior to harvesting, the cells were treated with cycloheximide (100 μg/ml) for 15 min at 37°C. The cells were then washed once in ice-cold 1X PBS containing 100 μg/ml cycloheximide and then lysed in homogenization buffer [15 mM HEPES–KOH (pH 7.4), 7.5 mM MgCl_2_, 100 mM KCl, 2 mM DTT, 1% Triton X-100, 100 μg/ml cycloheximide, 1X protease inhibitor cocktail and 20 U/ml RNAsin]. Cell lysates were triturated by passing them 5 times through a 26G needle. The lysates were then spun at 12 000 rpm for 5 min and the supernatant was collected. The samples were normalized for equal amounts of RNA and sample volumes were equalized with the homogenization buffer. The samples were then loaded onto a linear sucrose density gradient (5%-50% w/w prepared in solution containing 15 mM HEPES–KOH, 7.5 mM MgCl_2_, 100 mM KCl, 2 mM DTT, 100 μg/ml cycloheximide using BioComp gradient master, BioComp Instruments) and subjected to ultracentrifugation (36 000 rpm, 2 h, 4°C, Beckman SW41 rotor, Beckman LS-80M Ultracentrifuge). The fractionated samples were then analysed, and the profiles were recorded using the BioComp Gradient Fractionator. For polysome/monosome ratio the area under the peaks was measured using OriginPro software.

### [^35^S]-Met incorporation assay

Control and SIRT6 stable knockdown HEK 293T cells were grown in 6-well plates. Prior to labelling, the media was replaced with methionine-free DMEM for 2 h. Labelling of newly synthesized proteins was carried out using 100 mCi/well of ^35^S-In vivo Pro Twin label mix for 30 min. The cells were then directly lysed either in 2X Laemmli buffer with β-mercaptoethanol and an equal amount of protein from each sample were loaded onto the gels. The proteins were then transferred onto a PVDF membrane and then subjected to autoradiography using phosphor imager. Parallelly, a duplicate gel was run and was stained with Coomassie stain to check equal protein loading.

### [^3^H]-Leucine incorporation assay

The cardiomyocytes transfected with either control or SIRT6 siRNA for 48 h were incubated with [^3^H]-Leucine in leucine-free DMEM medium for 24 h. For induction with IGF-1, the cells were treated with 20 ng/ml of IGF-1 for 24 h. Cells were washed with 1X PBS and then incubated in 10% trichloroacetic acid to precipitate proteins. The resultant pellet was solubilized in 0.2 N NaOH and diluted with one-sixth volume of scintillation fluid and counted in a scintillation counter. Values were normalized with DNA content, which was measured using the Qubit dsDNA HS assay kit.

### Chromatin Immunoprecipitation (ChIP)

ChIP was performed using the SimpleChIP^®^ Enzymatic Chromatin IP Kit as per manufacturers protocol. Briefly, cells were grown in 150 mm dishes until 70–80% confluence and cross-linking was performed for 10 min with 1% formaldehyde and subsequently quenched with 0.125 M glycine. The cells were scraped in 1X ice-cold PBS, collected by centrifugation and resuspended in buffer. The chromatin was enzymatically digested using micrococcal nuclease to around 150–600 bp. The DNA–protein complexes were then pulled down with the specific antibodies. Magnetic beads provided in the kit were used to pellet the immunoprecipitated complexes. The samples were then decrosslinked, and the total DNA was extracted and analysed by quantitative real-time PCR.

### Reporter gene assays

Luciferase reporter assays were performed using the Dual Luciferase Reporter Assay System from Promega according to manufacturer instructions. For monitoring cap-dependent translation the cells were transfected with the pRL-CMV construct along with the indicated plasmids. Twenty four hours post transfection the cells were trypsinized and distributed into two halves. One half was used for isolation of RNA and subsequent cDNA preparation, while the other half was utilized for lysate preparation. The luciferase activity from the lysate was recorded using a standard luminometer. The *Renilla* luciferase readings were then normalized against the mRNA levels of *Renilla* luciferase gene quantified by qPCR (GAPDH was used as an internal control) and also for equal amounts of protein.

### Real-time PCR

Total RNA was extracted from the cells using the TRI-reagent according to manufacturer's protocol. RNA was checked for integrity and 1 μg of RNA was reverse transcribed and qPCR was performed using SYBR-Green PCR master mix in a real-time PCR system.

### Immunofluorescence microscopy

The cells were plated on sterilized glass cover slips placed inside a 12-well plate. The cells were fixed after indicated treatments using 3.7% formaldehyde for 10–15 min at room temperature. Permeabilization was carried out with 0.2% Triton X-100 for 5 min at room temperature followed by incubation with blocking buffer (1% BSA in PBS containing 22.52 mg/ml glycine and 0.1% Tween 20) for 45 min. The cells were then incubated overnight at 4°C with indicated primary antibodies prepared in the blocking buffer at dilutions ranging from 1:100–1:300. The cells were then incubated with species specific Alexa Fluor conjugated secondary antibodies for 1 h at room temperature. The nuclei were stained with Hoechst 33342 by incubation for 5 min at room temperature. The cells were washed twice with 1X PBS between each step. Finally, the cells were mounted on a clean glass cover slide using the Fluoromount-G Aqueous Mounting Medium and the images were acquired using Zeiss LSM 710 or 880 confocal microscope.

### Protein purification

Sp1 zinc-finger DNA binding domain (ZFDBD) was cloned in pET28a vector with an N-terminal 6X-His tag. Proteins were expressed in *Escherichia coli* BL21(DE3)-Rosetta cells and induced by using 1 mM IPTG and 0.1 mM ZnSO_4_ at 20°C. After 16 h of induction, cells were harvested and resuspended using Buffer A (8 mM Na_2_HPO_4_, 1.5 mM KH_2_PO_4_, 2.7 mM KCl, 137 mM NaCl and 1 mM DTT). The lysate was loaded on a Mono S HR column and the protein was eluted by a gradient of NaCl (0 to 1M). The protein was further purified by gel-filtration chromatography using Buffer B (10 mM Tris–HCl, pH 8, 150 mM NaCl and 1 mM DTT). The eluted protein fractions were combined and dialyzed against a buffer consisting of 10 mM Tris–HCl, pH 7.4, 50 mM NaCl and 1 mM DTT. Uniformly isotope-labelled protein (^15^N) was expressed in *E. coli* grown in M9 media using ^15^NH_4_Cl as the sole source of nitrogen.

His tagged wild-type SIRT6 cloned in pET28a vector was obtained from Addgene (Addgene no: 28271). The H133Y mutant was generated by site-directed mutagenesis of the wild-type SIRT6 construct. SIRT6-WT and SIRT6-H133Y were expressed in *E. coli* BL21(DE3)-Rosetta cells using 1 mM IPTG for induction at 20°C. The proteins were purified using His-tag affinity chromatography followed by gel-filtration chromatography under a buffer consisting of: 50 mM Tris, pH 7, 150 mM NaCl, and 1 mM DTT. Protein was exchanged against a buffer consisting of 10 mM Tris–HCl, pH 7.4, 50 mM NaCl and 1 mM DTT.

### NMR titration of ^15^N Sp1 zinc finger domain with SIRT6-WT and SIRT6-H133Y mutant

Uniformly ^15^N labelled Sp1 ZFDBD was titrated with increasing concentration of SIRT6-WT or mutant SIRT6-H133Y till a final ratio of 1:2 (Sp1 ZFDBD to SIRT6-WT or SIRT6-H133Y) and a 2D ^15^N–^1^H TROSY HSQC NMR spectra was recorded at each step of titration. NMR titration spectra were recorded on a BRUKER 700 MHz NMR spectrometer equipped with HCN cryoprobe at 25°C. NMR data were recorded using Topspin 3.1. Datasets were processed using Topspin3.1 and analysed using the SPARKY software (UCSF, CA, USA). The resonance assignment and the residue numbering of Sp1 ZFDBD was taken from a previously published study (20).

### Bioinformatic analysis

Overlaps between mTOR gene sets and transcription factor gene sets were computed using the MSigDB v6.2. The analysis of the ChIP-seq datasets was carried out as described previously with certain modifications (21). Processed ChIP-seq datasets for SIRT6 and Sp1 from K562 and hESC1 cell lines (hg19 alignment) were obtained from ENCODE database. Transcription start site (TSS) co-ordinate file was obtained from the UCSC table browser (22). ‘Slop’ tool from Bedtools package (v2.25.0-Quinlan, 2014) was used to analyse the transcription factor occupancy at the region around TSS (3 kb extension for SIRT6 and 1 kb extension for Sp1). ‘IntersectBed’ tool from BedTools was used to identify genes that were bound by SIRT6 within 3 kb and Sp1 within 1 KB of TSS. R (v 3.2.3) package ‘Vennerable’ (Version 3.0- http://rforge.rproject.org/projects/vennerable) was used to create the Venn diagram to depict the overlap between SIRT6 and Sp1 binding regions. The binding of SIRT6 and Sp1 in the promoters of mTOR signalling genes was visualized through their signal intensities using the integrated genome viewer (IGV). Enrichment for the mTOR signalling pathway in the Sp1 ChIP dataset was computed using the GSEA tool (23).

Promoter analysis of mTOR signalling genes was performed using tools from the Genomatix Software Suite v3.6. Gene promoters corresponding to coding transcripts for various organisms were retrieved from the EiDorado database and were aligned for conserved transcription factor binding sites, using the DiAlign program (Both EiDorado and DiAlign are offered as part of Genomatix Software Suite). The results were further refined by performing a local alignment of the Sp1 binding regions using the Clustal Omega server from the EBI. Primers for ChIP-qPCR experiments were designed in the Sp1 binding regions of the promoter sequences using the NCBI Primer Blast server.

### Key resources table

The list and the details of all the key resources used in the study including chemicals, reagents, antibodies, plasmids and primers are presented in Supplemental file 3.

### Quantification and statistical analysis

Statistical analysis and graph preparation were done using Graph-pad prism version 6.04. *t*-test was used for pair-wise comparisons. One-way ANOVA and two-way ANOVA were used for multiple comparisons. ZEN-Black software was used for confocal image analysis and Image Studio Lite Ver 5.2 was used for quantification of western blots. OriginPro was used to calculate the area under the curve for the polysome profiles.

## RESULTS

### SIRT6 deficiency leads to increased global protein synthesis levels

**Figure 1.**
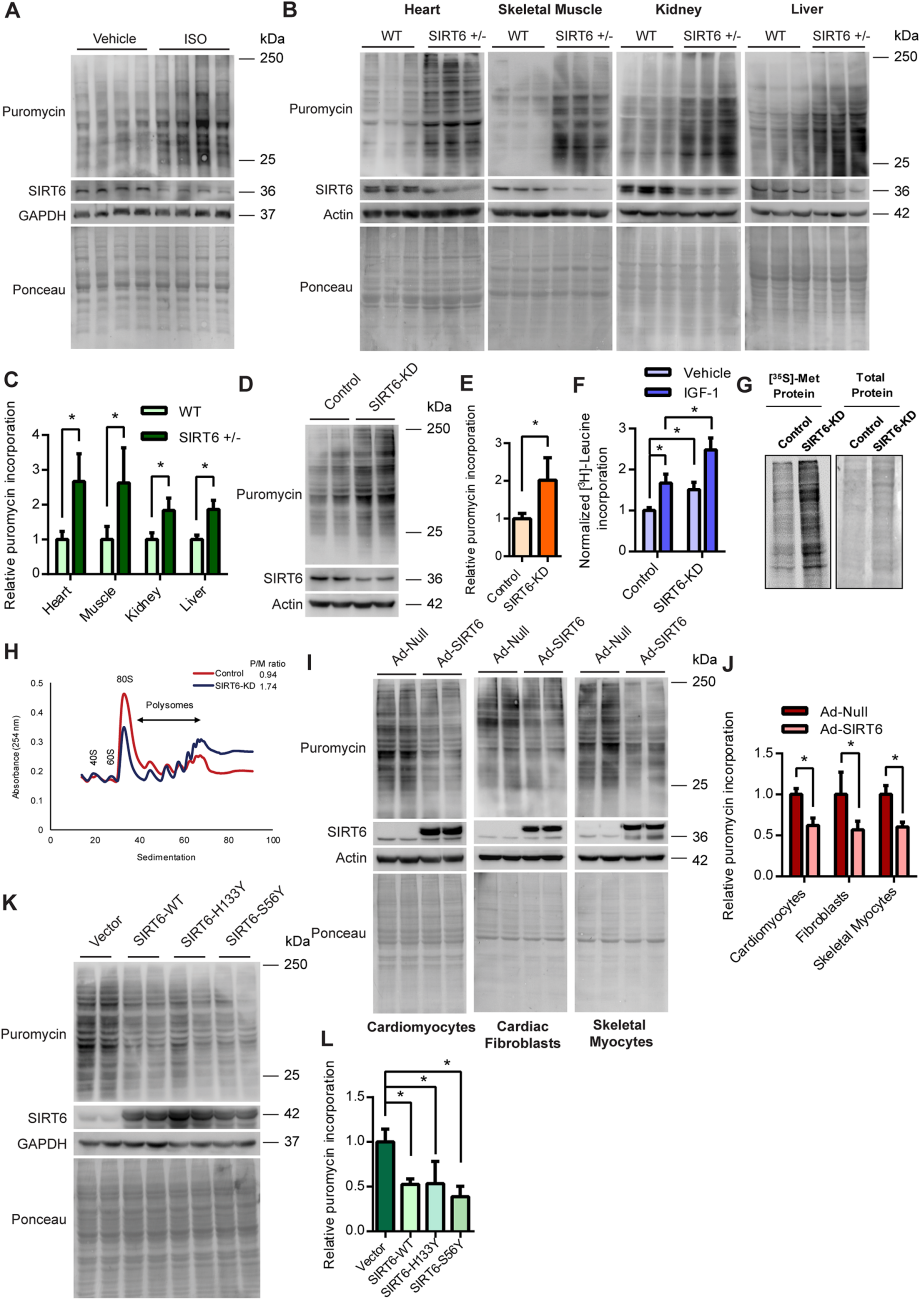
(**A**) Western blotting analysis depicting the changes in protein synthesis rates (tracked using SUnSET assay) and concomitant changes in SIRT6 levels in heart tissue of ISO administered mice. (**B**) Representative images of western blotting SUnSET analysis depicting changes in protein synthesis rates in heart, skeletal muscle, kidney and liver tissues of WT and SIRT6 heterozygous (SIRT6 +/–) mice. (**C**) Quantitative representation of puromycin incorporation depicted in Figure 1B. The results are expressed as the fold change relative to wild-type controls. *n* = 6 mice per group. Data are presented as mean ± s.d, *P < 0.05. (**D**) Representative images of western blotting SUnSET analysis of control and SIRT6-depleted (SIRT6-KD) neonatal rat cardiomyocytes. SIRT6 was depleted using specific siRNA and the knockdown was confirmed by immunoblotting for SIRT6. (**E**) Quantitative representation of puromycin incorporation depicted in Figure 1D. The results are expressed as the fold change relative to control cells. *n* = 5. Data are presented as mean ± s.d, *P < 0.05. (**F**) Protein synthesis rates assessed by [^3^H]-leucine incorporation in control or SIRT6-KD neonatal rat cardiomyocytes in the presence of vehicle or 20 ng/ml of IGF-1. [^3^H]-leucine incorporation was normalized against the DNA content. The results are expressed as the fold change relative to vehicle-treated control cells. *n* = 6. Data are presented as mean ± s.d, *P < 0.05. (**G**) Representative images of protein synthesis rates observed using [^35^S]-Methionine incorporation in control and SIRT6 stable knockdown (SIRT6-KD) 293T cells. Coomassie staining was done to confirm equal loading. (H) Polysome profiles of control and SIRT6 stable knockdown 293T cells indicating the cellular translation rates. The polysome to monosome ratio was calculated by measuring the area under the curves representing the respective fractions. The profiles and the ratio presented here are representative of three independent experiments. SIRT6 knockdown was confirmed by western blotting and is shown in Supplemental Figure S1B. (**I**) Representative images of western blotting SUnSET analysis of null or SIRT6 adenovirus-infected neonatal rat cardiomyocytes, cardiac fibroblasts or skeletal myocytes. The cells were infected with adenovirus for 48 h and the expression of SIRT6 was confirmed by immunoblotting. (**J**) Quantitative representation of puromycin incorporation shown in Figure 1I. The results are expressed as the fold change relative to respective null adenovirus-infected cells. *n* = 4–5. Data are presented as mean ± s.d, *P < 0.05. (**K**) Representative images of western blotting SUnSET analysis in 293T cells transfected with pcDNA, SIRT6-WT, SIRT6-H133Y or SIRT6-S56Y plasmids. The plasmids were transfected for 48 h and the expression of SIRT6 and its catalytic mutants were confirmed by western blotting. (**L**) Quantitative representation of puromycin incorporation depicted in Figure 1K. The results are expressed as the fold change relative to respective pcDNA transfected cells. *n* = 4. Data are presented as mean ± s.d, *P < 0.05.

We have previously reported that the rate of protein synthesis is increased in heart during isoproterenol (ISO) induced cardiac hypertrophy (18). In the present study, we found that the levels of SIRT6 were reduced in the ISO-induced hypertrophic hearts, concomitant with the increase in global protein synthesis (Figure 1A, Supplemental Figure S1A). We therefore hypothesized that SIRT6 might play a role in the regulation of protein synthesis. To test this, we compared the *in vivo* protein synthesis rates in different tissues of wild-type and SIRT6 heterozygous mice using the puromycin incorporation based SUnSET assay (17,18). Interestingly, we observed that the protein synthesis rates were significantly upregulated in multiple tissues of SIRT6 heterozygous mice including the heart, skeletal muscle, kidney and liver as compared to the wild-type controls, indicating that SIRT6 deficiency leads to enhanced protein synthesis (Figure 1B, C). To determine whether the observed effects on protein synthesis occur in a cell-autonomous fashion, we either depleted or overexpressed SIRT6 in multiple cell types and evaluated the changes in protein synthesis. We observed that the protein synthesis rates were markedly upregulated, when SIRT6 was transiently depleted using a specific siRNA (Figure 1D, E). Further, we observed that [^3^H]-leucine incorporation was significantly enhanced in SIRT6 depleted primary cardiomyocytes under basal as well as IGF-1 treated conditions (Figure 1F). Similarly, we observed increased [^35^S]-methionine incorporation, when we assessed protein synthesis in SIRT6 depleted cells (Figure 1G). Next, we performed polysome-profiling to compare the cellular translation rates in control and SIRT6 knockdown cells. We found a relatively increased fraction of polysomes in SIRT6 depleted cells compared to control cells, suggesting that SIRT6 deficiency leads to increased translation (Figure 1H, Supplemental Figure S1B). While SIRT6 deficiency upregulates global protein synthesis, overexpression of SIRT6 suppressed protein synthesis in multiple cell types including cardiomyocytes, cardiac fibroblasts and skeletal myocytes (Figure 1I and J). Since SIRT6 is downregulated during cardiac hypertrophy (Figure 1A), we tested whether overexpression of SIRT6 can reduce the augmented cardiac protein synthesis induced by hypertrophic agonist phenylephrine. Immunofluorescence SUnSET analysis suggests that overexpression of SIRT6 markedly reduced the protein synthesis in cardiomyocytes treated with phenylephrine (Supplemental Figure S1C). These results indicate that SIRT6 negatively regulates protein synthesis in cells under diverse conditions.

To test whether the regulation of protein synthesis by SIRT6 is dependent upon its catalytic activity, we assessed the protein synthesis rates in cells transfected with wild-type or catalytic mutants of SIRT6 (H133Y or S56Y). Both the mutants SIRT6-H133Y and SIRT6-S56Y have been reported to lack the deacetylase as well as ADP ribosyl transferase activity (24,25). Surprisingly, we observed that, in addition to wild-type SIRT6, the catalytic mutants also markedly attenuated the protein synthesis rates (Figure 1K, L), suggesting that SIRT6 might regulate protein synthesis independent of its catalytic activity.

### SIRT6 controls protein synthesis through the mTOR signalling

**Figure 2.**
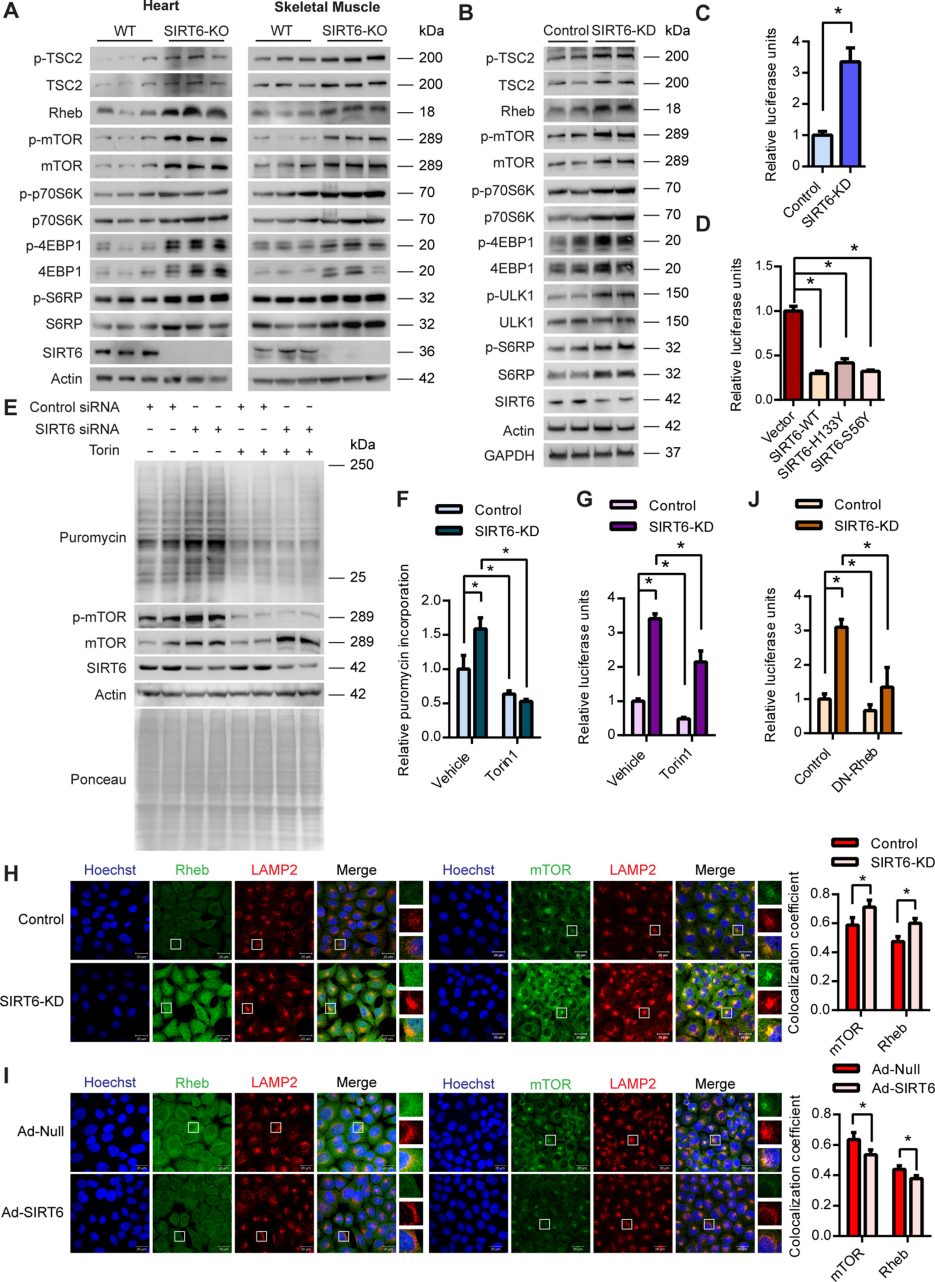
(**A**) Representative images of western blotting analysis of mTOR signalling in the heart and skeletal muscle lysates of WT and SIRT6-KO mice. *n* = 6–7 mice per group. (**B**) Representative images of western blotting analysis of mTOR signalling genes in control and SIRT6 stable knockdown 293T (SIRT6-KD) cell line. (**C**) Quantitative representation of cap-dependent translation rates measured using the luciferase reporter construct pRL-CMV in control and SIRT6 stable knockdown 293T (SIRT6-KD) cells. *Renilla* luciferase readings were normalized against the mRNA levels of *Renilla* luciferase. *n* = 6. Data are presented as mean ± s.d, *P < 0.05. (**D**) Quantitative representation of cap-dependent translation rates measured using the luciferase reporter construct pRL-CMV in HEK 293T cells transfected with pcDNA, SIRT6-WT, SIRT6-H133Y or SIRT6-S56Y plasmids. *Renilla* luciferase readings were normalized against the mRNA levels of *Renilla* luciferase. *n* = 5. Data are presented as mean ± s.d, *P < 0.05. (**E**) Representative images of western blotting SUnSET analysis in control or SIRT6-depleted (SIRT6-KD) HeLa cells in the presence or absence of the 250nM of mTOR inhibitor Torin1. DMSO was used as vehicle control. SIRT6 was depleted using specific siRNA and the knockdown was confirmed by immunoblotting for SIRT6. Reduction in mTOR phosphorylation was used to confirm the action of Torin1. (**F**) Quantitative representation of puromycin incorporation shown in Figure 2E. The results are expressed as the fold change relative to control cells. *n* = 4. Data are presented as mean ± s.d, *P < 0.05. (**G**) Quantitative representation of cap-dependent translation rates measured using the luciferase reporter pRL-CMV in control or SIRT6 depleted HeLa cells (siRNA mediated knockdown) in the presence or absence of 250 nM of Torin1. *Renilla* luciferase readings were normalized against the mRNA levels of *Renilla* luciferase. *n* = 4. Data are presented as mean ± s.d., *P < 0.05. (**H**) Representative immunofluorescence images depicting the changes in the extent of co-localization of mTOR and Rheb with the lysosomes in control and SIRT6 depleted (SIRT6-KD) HeLa cells. mTOR and Rheb were stained green while the lysosomes marked with LAMP2 were stained red. SIRT6 was depleted using specific siRNA and knockdown was confirmed by immunostaining for SIRT6 (green) in a parallel experiment (Supplemental Figure S1E). The nuclei were stained with Hoechst 33342 and are shown in blue in all the panels. Scale bar = 20 μm. The colocalization was quantified using Zen Software Suite and the values are represented relative to control cells. *n* = 50–100 cells per group. (**I**) Representative immunofluorescence images depicting the changes in the extent of co-localization of mTOR and Rheb with the lysosomes in null or SIRT6 adenovirus-infected HeLa cells. mTOR and Rheb were pseudo-coloured green while the lysosomes marked with LAMP2 were pseudo-coloured red. The cells were infected with adenovirus for 48 h and SIRT6 overexpression was confirmed by immunostaining for SIRT6 (green) in a parallel experiment (Supplemental Figure S1F). The nuclei were stained with Hoechst 33342 and are shown in blue in all the panels. Scale bar = 20 μm. The colocalization was quantified using Zen Software Suite and the values are represented relative to null adenovirus-infected cells. *n* = 50–100 cells per group. (**J**) Quantitative representation of cap-dependent translation rates measured using the luciferase reporter pRL-CMV in control or SIRT6 depleted HeLa cells (siRNA mediated knockdown) under control or Rheb inhibited conditions. For Rheb inhibition dominant negative Rheb D60K mutant (DN-Rheb) was ectopically expressed in the cells. *Renilla* luciferase readings were normalized against the mRNA levels of *Renilla* luciferase. *n* = 3. Data are presented as mean ± s.d, *P < 0.05.

The mTOR signalling pathway is the master regulator of protein synthesis in the cells. Under favourable and nutrient-rich conditions, activation of the mTORC1 complex by its upstream activator Rheb leads to stimulation of the protein synthesis in the cell through phosphorylation of its downstream targets p70S6K and 4EBP1 (13). We therefore tested whether SIRT6 possibly regulates the cellular protein synthesis through the mTOR signalling axis. To decipher the role of SIRT6 in the regulation of mTOR signalling, we measured the total and phosphorylated protein profiles of mTOR and its downstream targets in control and SIRT6 knockout mice. We observed that the phosphorylation of mTOR as well as its downstream targets p70S6K, 4EBP1 and S6 ribosomal protein (S6RP) were markedly upregulated in heart and skeletal muscle of SIRT6-KO mice, indicating augmented mTOR signalling under SIRT6 deficient conditions (Figure 2A). Interestingly, we observed that the total protein levels of the key activators of the mTOR pathway such as the Rheb, mTOR, p70S6K and S6RP were also markedly upregulated in SIRT6 knockout mice (Figure 2A). Though, the levels of negative regulators such as TSC2 and 4EBP1 were high under SIRT6 knockout conditions, their inhibitory phosphorylation levels were also high, suggesting a net activation of the mTOR pathway. Further, in line with the *in vivo* evidence, we observed increased total as well as phospho-protein levels of mTOR signalling genes in SIRT6 knockdown cells (Figure 2B). We find that the phosphorylation of ULK1 was increased upon SIRT6 depletion, while the total levels of ULK1 were not changed (Figure 2B). This critical finding establishes the fact that mTOR signalling is hyperactive in SIRT6 deficient cells (Figure 2B).

The activation of mTOR is well correlated with an increase in the 5′ cap-dependent mRNA translation (26,27). We therefore examined the cap-dependent translation under *in vitro* conditions using the luciferase reporter construct, pRL-CMV, which generates 5′ capped luciferase transcripts. The luciferase activity normalized to the transcript levels of the luciferase gene served as a measure of cap-dependent translation in the cells. Consistent with the increase in global translation, SIRT6 depleted cells displayed increased cap-dependent translation (Figure 2C). Conversely, SIRT6 overexpressing cells displayed diminished cap-dependent translation compared to control cells (Figure 2D). Further, we observed that overexpression of wild-type SIRT6, as well as its catalytic mutants, markedly suppressed cap-dependent translation (Figure 2D), reaffirming that SIRT6 regulates protein translation independent of its catalytic activity. These results suggest that the changes in global translation due to alterations in SIRT6 levels may be attributed at least in part to changes in cap-dependent translation, which is controlled by the mTOR signalling pathway.

We next tested whether increased protein synthesis observed under SIRT6 deficient conditions could be rescued by the inhibition of mTOR signalling. We observed that inhibition of mTOR activity by rapamycin or ATP competitive inhibitor Torin1 indeed abrogated the increased protein synthesis observed in SIRT6 depleted cells (Figure 2E, 2F and Supplemental Figure S1D). Further, we observed that inhibition of mTOR with Torin1 significantly suppressed the cap-dependent translation observed in SIRT6 depleted cells (Figure 2G). However, unlike the results from the SUnSET assay, the relative cap-dependent translation rates were still higher in SIRT6 depleted cells compared to control cells in the Torin1 treated group. This is due to important differences in the nature of the two techniques employed to assess protein synthesis. While SUnSET assay measures protein synthesis during a brief time window of 30 min following Torin1 treatment, the cap dependent reporter activity represents the translation rate over several hours extending even beyond the Torin1 treatment time window. Nevertheless, these results strongly indicate that SIRT6 controls protein synthesis through the mTOR signaling pathway.

We next asked how increased levels of mTOR and Rheb could activate mTOR signalling in SIRT6-deficient conditions. A key step in the activation of mTOR signalling is the recruitment of the mTORC1 complex to the surface of lysosomes, where the upstream GTPase activator Rheb, stimulates mTOR activity (28,29). We therefore evaluated the extent of colocalization of mTOR and Rheb to lysosomes in control and SIRT6 knockdown cells by confocal microscopy. In line with the increased total protein levels, we observed enhanced colocalization of mTOR and Rheb with lysosomes in SIRT6 depleted cells (Figure 2H, Supplemental Figure S1E). Intriguingly, we observed increased levels of lysosomes under SIRT6 deficient conditions (Figure 2H), which we are currently investigating in a separate study. Conversely, overexpression of SIRT6 led to a marked reduction in the colocalization of mTOR and Rheb with lysosomes (Figure 2I, Supplemental Figure S1F). To test whether the increased Rheb levels contribute to the activation of mTOR, we inhibited endogenous Rheb by ectopically expressing a dominant negative Rheb construct and tested the cap-dependent translation. We found that inhibition of Rheb significantly reduced the cap-dependent translation observed in SIRT6 depleted cells (Figure 2J). These findings suggest that increased protein levels and localization of Rheb and mTOR to lysosomes could lead to the increased mTORC1 activity under SIRT6 deficient conditions.

### SIRT6 transcriptionally regulates mTOR signalling

**Figure 3.**
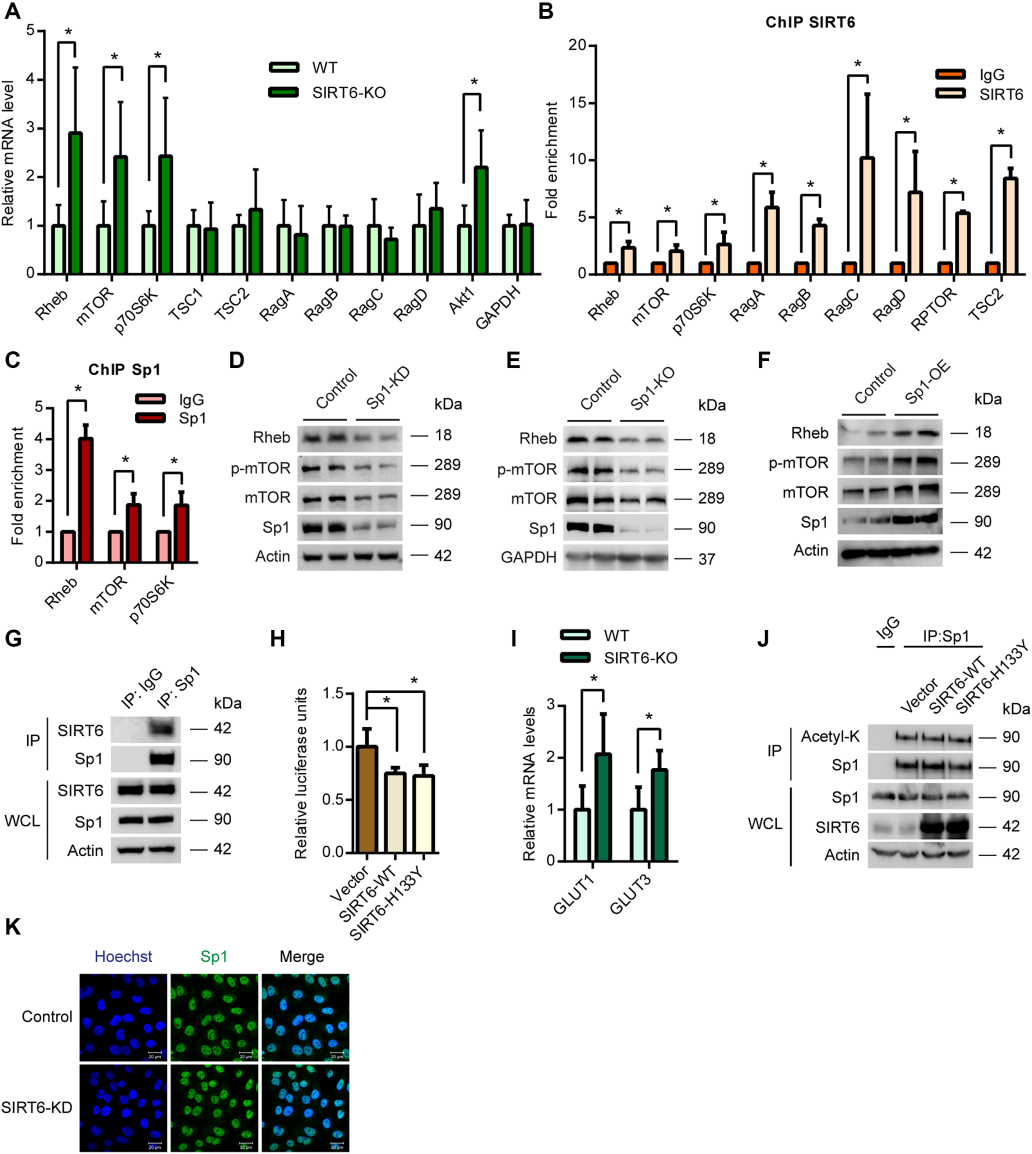
(**A**) qPCR analysis of relative mRNA expression levels of mTOR signalling genes in WT and SIRT6-KO mice heart tissues. *n* = 4–10 mice per group. Data are presented as mean ± s.d, *P < 0.05. AKT1 was used as a positive control. GAPDH was used as a negative control. (**B**) ChIP analysis to detect SIRT6 binding to the promoters of the indicated genes performed with a SIRT6-specific antibody or IgG control antibody in WT 293T cells. *n* = 3–4. Data are presented as mean ± s.d, *P < 0.05. (**C**) ChIP analysis to detect Sp1 binding to the promoters of the indicated genes performed with a Sp1-specific antibody or IgG control antibody in WT 293T cells. *n* = 3–4. Data are presented as mean ± s.d, *P < 0.05. (**D**) Representative images of western blotting analysis of mTOR signalling under Sp1 depleted (Sp1-KD) conditions in 293T cells. The Sp1 levels were depleted by transfecting the cells with specific shRNA and the knockdown was confirmed by western blotting. (E) Representative images of western blotting analysis of mTOR signalling in Sp1-KO HeLa cells. Sp1 was deleted using a specific guide RNA and the CRISPR-Cas9 system. (**F**) Representative images of western blotting analysis of mTOR signalling under Sp1 overexpression conditions in 293T cells. The cells were transfected with empty vector or Sp1 expressing plasmid for 48 h and Sp1 overexpression was confirmed by immunoblotting. (**G**) Sp1 was immunoprecipitated from 293T cells and the interaction with SIRT6 was tested by western blotting. IgG was used as control. Whole cell lysate (WCL) was probed for indicated proteins by western blotting. The results presented here are representative of three independent experiments. (**H**) Luciferase reporter assay to assess the transcriptional activity of Sp1 was performed in pcDNA, SIRT6-WT or SIRT6 H133Y expressing 293T cells. The results are expressed as the fold change relative to pcDNA expressing cells. *n* = 6. Data are presented as mean ± s.d, *P < 0.05. (**I**) Realtime qPCR analysis of relative mRNA levels of GLUT1 and GLUT3 in WT and SIRT6-KO mice heart tissues. *n* = 6 mice per group. Data are presented as mean ± s.d, *P < 0.05. (**J**) Sp1 was immunoprecipitated from the 293T cells transfected with pcDNA, SIRT6-WT or SIRT6-H133Y plasmids and western blotting analysis was performed to detect the levels of Sp1 acetylation (Ac-Lys) by anti-acetyl-lysine antibody. IgG was used as a negative control in this assay. Whole cell lysate (WCL) was probed for indicated proteins by western blotting. (K) Representative immunofluorescence images depicting the levels and localization of Sp1 transcription factor (green) under control and SIRT6-KD conditions in Hela cells. SIRT6 was depleted using specific siRNA and knockdown was confirmed by immunostaining for SIRT6 (green) in a parallel experiment (Supplemental Figure S2E). The nuclei were stained with Hoechst 33342 and are shown in blue. Scale bar = 20 μm.

SIRT6 is a chromatin-associated protein previously known to regulate multiple signalling pathways at the transcriptional level (30–32). To test whether SIRT6 regulates the mTOR signalling at the transcriptional level, we assessed the mRNA levels of the key proteins of the mTOR signalling pathway. Realtime qPCR analysis revealed significant up-regulation of the transcript levels of key activators of mTOR signalling such as the Rheb, mTOR and p70S6K in SIRT6-KO mice heart tissues (Figure 3A). However, we did not observe any significant changes in the mRNA levels of other proteins influencing mTOR signalling such as the Rag GTPases (Figure 3A). Since SIRT6 is known to control the transcription by directly binding to the promoter regions, we tested whether SIRT6 binds to the promoters of key mTOR signalling genes. We performed ChIP assay and found significant enrichment of SIRT6 at the promoters of key mTOR signalling genes compared to IgG controls (Figure 3B). These findings indicate that SIRT6 directly binds to the promoters of mTOR signalling genes to control its transcription.

### Sp1 transcription factor regulates mTOR signalling

SIRT6 is known to repress the transcription of its target genes by interacting with various transcription factors such as HIF1α, NFκB, c-Myc, c-Jun and LEF1 (21,30,31,33,34). We envisaged that SIRT6 could regulate the mTOR pathway through a transcription factor that controls the expression of mTOR signalling genes. To predict the potential transcription factor that could regulate mTOR signalling, we computed the overlap between the mTOR pathway gene sets and a collection of transcription factor target gene sets (C3: TFT in MSigDB, 615 gene sets) available in the MSigDB database. We observed that multiple mTOR gene sets available in the MSigDB database displayed significant overlap with the TFT gene set GGGCGGR_SP1_Q6 which corresponds to the Sp1 transcription factor. Notably, the mTOR pathway gene set PID_MTOR_4PATHWAY displayed maximum enrichment for the GGGCGGR_SP1_Q6 gene set with a *P*-value of 2.77 × 10^−12^ (Supplemental file 1). In addition, gene set enrichment analysis indicated that the PID_MTOR_4PATHWAY gene set was enriched in the Sp1 ChIP-seq data set (Supplemental Figure S2A). Further, we observed that most of the other transcription factor gene sets, which showed comparable overlaps with the mTOR gene sets did not display binding to mTOR and/or other critical genes of the pathway (data not shown). We therefore proceeded with the Sp1 transcription factor for further analysis.

To gain insights on the mTOR signalling genes targeted by Sp1, we analysed the promoters of various genes of mTOR signalling using the Genomatix software suite. Our analysis revealed evolutionarily conserved Sp1 binding sites in the key genes of the pathway such as mTOR, Rheb and p70S6K (Supplemental file 2). We then performed ChIP analysis to experimentally verify the binding of Sp1 transcription factor to these promoters. We indeed found significant enrichment of Sp1 at the promoters of key mTOR signalling genes confirming the bioinformatic predictions (Figure 3C). We next tested whether Sp1 regulates the expression of mTOR signalling genes. Western blotting analysis revealed that transient depletion of Sp1 reduced the total protein levels of mTOR and Rheb (Figure 3D). We further observed reduced phosphorylation of mTOR in Sp1 depleted conditions (Figure 3D). To further confirm these findings, we generated stable Sp1 knockout cells using the CRISPR-Cas9 system. Similar to our knockdown experiments, we observed a reduction in the levels of mTOR and Rheb and reduced activation of mTOR (Figure 3E). On the contrary, overexpression of Sp1 increased the total protein levels of mTOR and Rheb and enhanced the phosphorylation of mTOR (Figure 3F). These findings confirm that Sp1 transcription factor plays a key role in the regulation of mTOR signalling.

### SIRT6 associates with the Sp1 transcription factor

To investigate a possible link between Sp1 and SIRT6, we compared the genome-wide binding maps of Sp1 and SIRT6 using the previously published ChIP-seq datasets (35–37). Interestingly, we observed that many of the genomic regions targeted by Sp1 were co-bound by SIRT6 (Supplemental Figure S2B, C). In line with this, analysis of the ChIP-Seq datasets from two independent cell lines (K562 and hESCs) (35–37), indicated that both SIRT6 and Sp1 bind to overlapping sites at the promoters of the mTOR signalling genes (Supplemental Figure S2D). Further, our immunoprecipitation experiments revealed that SIRT6 interacts with Sp1, indicating a potential association between the two molecules (Figure 3G).

To test whether SIRT6 regulates the activity of Sp1, we performed luciferase assay with a reporter plasmid containing Sp1 binding sites in the promoter. We observed that both wild-type, as well as the catalytically dead SIRT6, represses the promoter activity of Sp1 (Figure 3H). Notably, this correlates with the observed findings of SIRT6 on protein synthesis (Figures 1K, 1L and 2D). Since SIRT6 suppresses Sp1 transcription activity, we tested whether the effects of SIRT6 on Sp1 could extend beyond the mTOR signalling genes. To test this, we measured the transcript levels of two well-known Sp1 targets GLUT1 and GLUT3 (38,39) in SIRT6-KO mice. Interestingly, similar to mTOR signalling genes, we observed an upregulation of these Sp1 targets (Figure 3I), indicating that SIRT6 might regulate overall Sp1 transcription activity. SIRT6 has been previously reported to modulate the stability/activity of its interacting partners by affecting their acetylation status (40,41). We therefore tested whether SIRT6 deacetylates Sp1 transcription factor. Notably, we did not find any appreciable changes in the acetylation levels of Sp1 immunoprecipitated from cells transfected with WT or the catalytic mutants of SIRT6 (Figure 3J). We next checked whether SIRT6 influences the localization of Sp1 using confocal microscopy. However, we did not find any marked changes in the localization or levels of Sp1 in SIRT6 depleted cells (Figure 3K, Supplemental Figure S2E).

### SIRT6 binds to Sp1 and regulates its occupancy at mTOR signalling gene promoters

**Figure 4.**
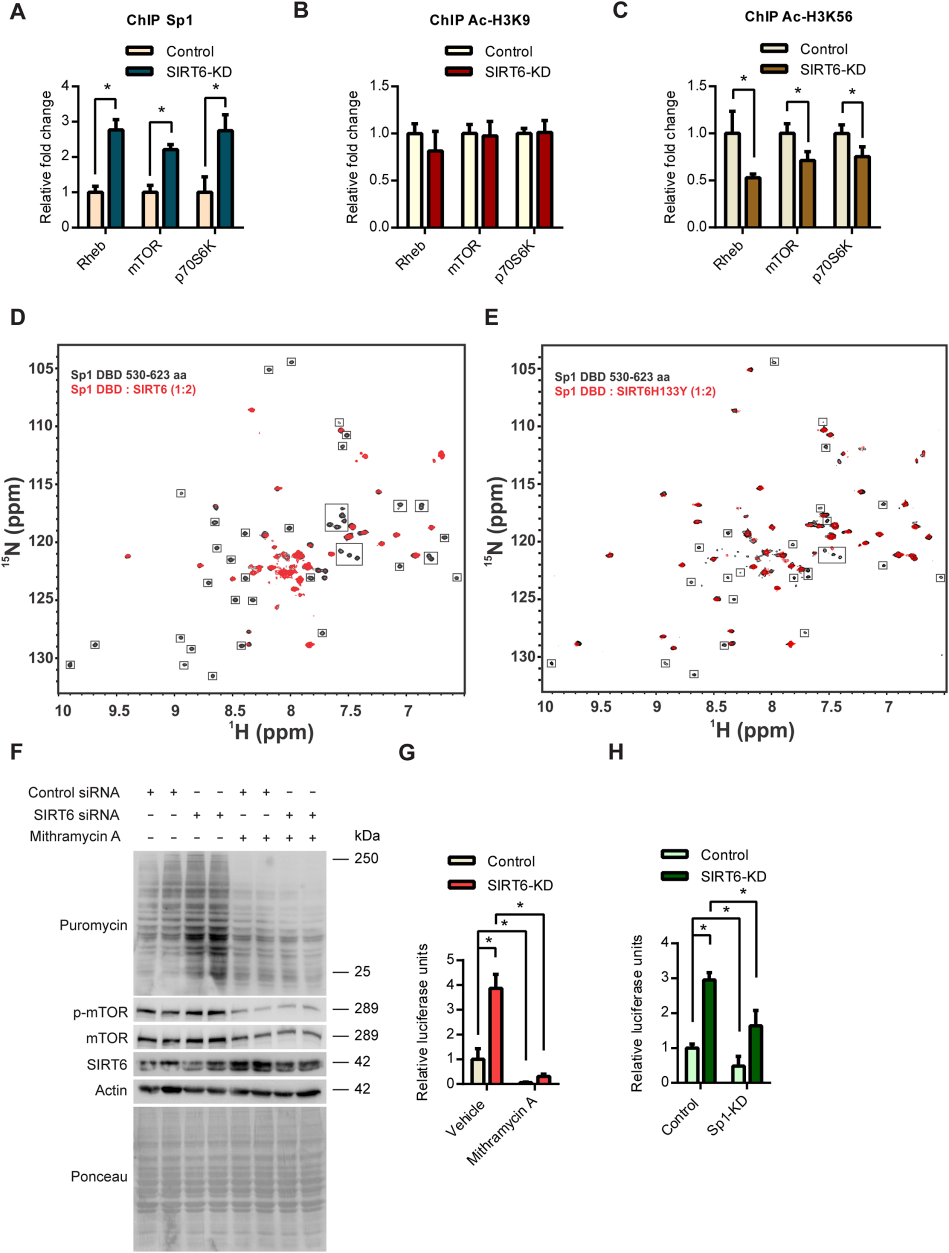
(**A**) ChIP analysis to detect changes in Sp1 binding to the Rheb, mTOR and p70S6K promoter regions in control or SIRT6 stable knockdown 293T cells performed with Sp1 or IgG control antibody. *n* = 3. Data are presented as mean ± s.d, *P < 0.05. (**B**) ChIP analysis to detect changes in acetylation status at H3K9 residue at Rheb, mTOR and p70S6K promoter regions in control or SIRT6 stable knockdown 293T cells performed with Sp1 or IgG control antibody. *n* = 3. Data are presented as mean ± s.d, *P < 0.05. (**C**) ChIP analysis to detect changes in acetylation status at H3K56 residue at Rheb, mTOR and p70S6K promoter regions in control or SIRT6 stable knockdown 293T cells performed with Sp1 or IgG control antibody. *n* = 3. Data are presented as mean ± s.d, *P < 0.05. (**D**) Overlay of 2D ^15^N-^1^H TROSY-HSQC NMR spectra of free Sp1 ZFDBD (black) with Sp1 ZFDBD in complex with SIRT6-WT (in red) (at 1:2 molar ratio). The disappeared cross peaks upon addition of SIRT6 are highlighted in box. (**E**) Overlay of ^15^N-^1^H TROSY-HSQC NMR spectra of free Sp1 ZFDBD (black) with Sp1 ZFDBD in complex with mutant SIRT6-H133Y (in red) (at 1:2 molar ratios). The disappeared cross peaks upon addition of mutant SIRT6-H133Y are highlighted in box. (**F**) Representative images of western blotting SUnSET analysis in control or SIRT6-KD HeLa cells in the presence vehicle or 200 nM of Sp1 inhibitor Mithramycin A. DMSO was used as a vehicle control. SIRT6 was depleted using specific siRNA and the knockdown was confirmed by immunoblotting for SIRT6. Reduction in mTOR phosphorylation was used to confirm the action of Mithramycin A. (**G**) Quantitative representation of cap-dependent translation rates measured using the luciferase reporter pRL-CMV in control or SIRT6 depleted HeLa cells (siRNA mediated knockdown) in the presence or absence of 200 nM Mithramycin A. *Renilla* luciferase readings were normalized against the mRNA levels of *Renilla* luciferase. *n* = 4. Data are presented as mean ± s.d, *P < 0.05. (**H**) Quantitative representation of cap-dependent translation rates measured using the luciferase reporter pRL-CMV in control or SIRT6 depleted HeLa cells (siRNA mediated knockdown) under control or Sp1 depleted conditions (shRNA mediated knockdown). *Renilla* luciferase readings were normalized against the mRNA levels of *Renilla* luciferase. *n* = 4. Data are presented as mean ± s.d, *P < 0.05.

Since SIRT6 is a chromatin-associated protein, we hypothesized that it may regulate the occupancy of Sp1 at the gene promoters. To test this possibility, we analysed the occupancy of Sp1 at the promoters of mTOR signalling genes in control and SIRT6 depleted cells. Interestingly, we observed increased occupancy of Sp1 at the promoters of Rheb, mTOR and p70S6K in SIRT6 depleted cells (Figure 4A). This indicates that SIRT6 might regulate the transcription of mTOR by controlling the occupancy of the Sp1 transcription factor at these promoters. SIRT6 is known to regulate the binding and recruitment of several factors by deacetylating histone residues H3K9 and H3K56 (42). We therefore tested whether SIRT6 influences these acetylation marks at the promoters of mTOR signalling genes. However, our ChIP analysis indicates that acetylation of these histone sites was not increased in SIRT6 depleted cells as compared to controls (Figure 4B and 4C).

Previous studies indicate that interaction with the zinc finger domain of Sp1 can interfere with the DNA binding activity of the protein (43). The Sp1 protein zinc finger DNA binding domain (ZFDBD) is made up of three C2H2-type zinc fingers (ZF1–3) which is the most highly conserved region among the Sp-family transcription factors (44). Although zinc fingers are mainly known for their DNA binding functions, they have also been implicated to play a key role in protein-protein interactions (45). Therefore, to test the hypothesis whether SIRT6 could directly interact with the ZFDBD of Sp1 and thereby likely affect its DNA binding function, we performed NMR spectroscopy based chemical shift perturbation experiments. We expressed and purified the uniformly ^15^N labelled ZFDBD of Sp1 and unlabelled SIRT6 (Supplemental Figure S3A). A 2D ^15^N–^1^H TROSY-HSQC NMR spectra of ZFDBD was recorded on a 0.2 mM sample at 25°C (Figure 4D). The spectra matched and overlaid well with the previously published spectra of Sp1 ZFDBD (20). The ^15^N labelled Sp1 ZFDBD was titrated with increasing concentration of unlabelled full-length SIRT6 up to final 1:2 molar ratio and a 2D ^15^N-1H TROSY-HSQC spectra was recorded at each step of titration (Figure 4D). We observed progressive broadening and loss in peak intensities of a set of cross peaks in Sp1 ZFDBD upon addition of SIRT6. At an over saturating 1:2 ratio of Sp1 ZFDBD to SIRT6, one set of resonance peaks disappeared in the 2D ^15^N-^1^H TROSY-HSQC spectra of the Sp1 ZFDBD (Figure 4D). This result clearly showed that Sp1 ZFDBD interacts with SIRT6 and likely the residues that lie at the interface of binding get exchange broadened due to intermediate exchange NMR timescale and therefore lost the signal intensity in the spectra.

Interestingly, a set of peaks in the spectra of Sp1 ZFDBD remained unperturbed and did not decrease in intensity upon SIRT6 titration. These residues likely belong to a region or subdomain of Sp1 ZFDBD that does not bind to SIRT6. We compared the spectra of Sp1 recorded here with the published spectra (20). Careful comparison showed that the majority of these resonances (which remained unperturbed upon SIRT6 binding) mapped to the zinc finger 3 (residues 596 to 623) of Sp1 ZFDBD (Supplemental Figure S3B). Therefore, the NMR chemical shift perturbation experiments suggest that the SIRT6 likely interacts with Sp1 ZFDBD using zinc finger 1 and zinc finger 2 of Sp1 ZFDBD. We also expressed and purified unlabelled mutant SIRT6 H133Y (Supplemental Figure S3C). The ^15^N labelled Sp1 ZFDBD was titrated with unlabelled full-length SIRT6 H133Y mutant with the final ratio of 1:2. A 2D ^15^N–^1^H TROSY-HSQC spectra of Sp1 ZFDBD was recorded at each step of titration (Figure 4E). We observed chemical shift perturbations and broadening of a number of resonances in the spectra of Sp1 ZFDBD which indicate that mutant SIRT6-H133Y also interacts with Sp1 ZFDBD. However, compared to the wild-type SIRT6-Sp1 ZFDBD titrations, only a subset of peaks was perturbed and disappeared. Some peaks that had disappeared in case of wild-type SIRT6-Sp1 ZFDBD titrations likely due to intermediate exchange, showed chemical shift perturbations in the fast exchange time scale. Overall, this suggests that mutant SIRT6-H133Y interacts with the Sp1 ZFDBD with some loss of binding compared to the wild-type SIRT6. These findings indicate that SIRT6 may regulate Sp1 transcriptional activity by directly binding to Sp1 ZFDBD, which may not require its catalytic activity.

Having confirmed the role of Sp1 in the SIRT6 mediated regulation of mTOR signalling, we next tested whether inhibition of Sp1 could reverse the increased protein synthesis observed in SIRT6 deficient cells. Interestingly, we observed that inhibition of Sp1 with the small molecule inhibitor, Mithramycin A attenuated the protein synthesis rates observed in SIRT6 deficient cells (Figure 4F). Similarly, we observed that inhibition or depletion of Sp1 reduced the increased cap-dependent translation observed under SIRT6 knockdown conditions (Figure 4G and 4H). Collectively, these findings suggest that SIRT6 represses Sp1 transcription factor to control the expression mTOR signalling genes and regulate protein synthesis.

### Inhibition of mTOR signalling improves cardiac function in muscle-specific SIRT6 knockout mice

**Figure 5.**
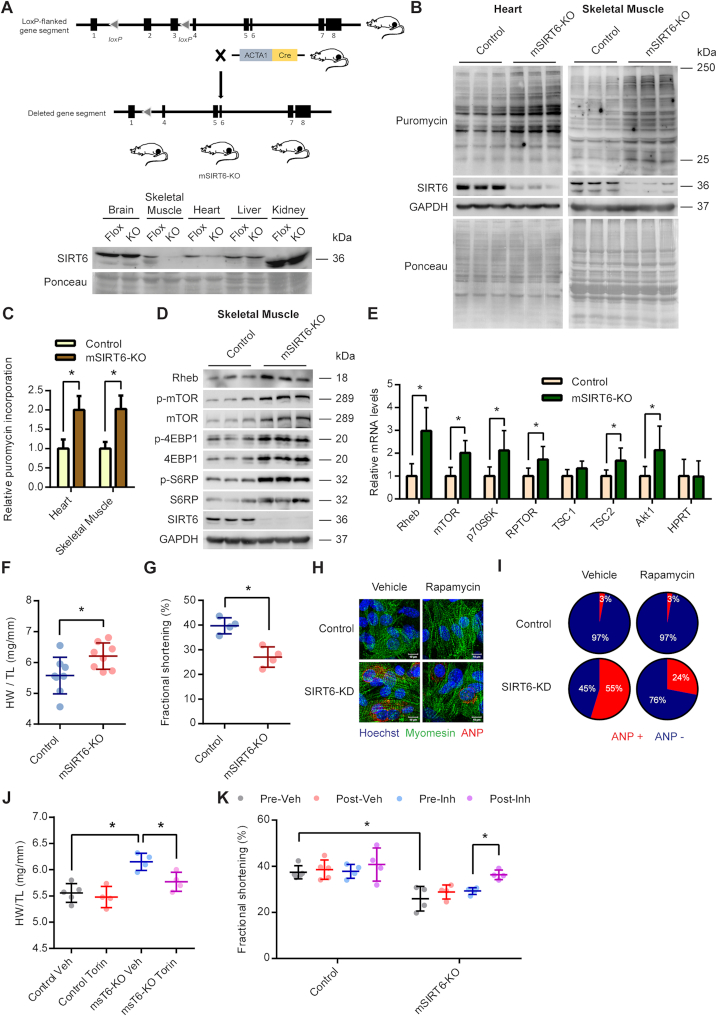
(**A**) Schematic explaining the generation of muscle-specific SIRT6 knockout mice (mSIRT6-KO) using the ACTA-Cre and SIRT6 floxed mice. Western blotting analysis was performed to confirm the deletion of SIRT6 in heart and skeletal muscle of the knockout mice. SIRT6-Flox mice were used as controls. (**B**) Representative images of western blotting SUnSET analysis depicting changes in protein synthesis rates in skeletal muscle and heart of control and mSIRT6-KO mice. (**C**) Quantitative representation of puromycin incorporation in heart and skeletal muscle shown in Figure 5B. The results are expressed as the fold change relative to SIRT6 floxed controls. *n* = 6 mice per group. Data are presented as mean ± s.d, *P < 0.05. (**D**) Representative images of western blotting analysis of mTOR signalling in skeletal muscle lysates of control and mSIRT6-KO mice. *n* = 6 mice per group. (E) qPCR analysis of relative mRNA expression levels of mTOR signalling genes in skeletal muscle tissues of control and mSIRT6-KO mice. *n* = 4–7 mice per group. Data are presented as mean ± s.d, *P < 0.05. AKT1 was used as a positive control. HPRT was used as a negative control. (F) Heart weight to tibia length ratio (HW/TL) of age-matched control and mSIRT6-KO mice at 9 months of age. *n* = 8 mice per group, Data are presented as mean ± s.d, *P < 0.05. (**G**) Echocardiographic analysis of cardiac function in age-matched control and mSIRT6-KO mice at 9 months of age. Fractional shortening was measured as an indicator of cardiac function. *n* = 4 mice per group, Data are presented as mean ± s.d, *P < 0.05. (H) Representative immunofluorescence images showing ANP staining in control and SIRT6 depleted (SIRT6-KD) primary cardiomyocytes treated with vehicle or 100 nM Rapamycin for 24 h. ANP was stained red and was used as a marker of hypertrophy. Myomesin was stained green and was used as a marker for cardiomyocytes. The nuclei were stained blue with Hoechst 33342. Scale bar = 20μm. (**I**) The percentage of cells showing ANP staining in each group shown in Figure 5H was calculated and represented as a pie-chart. (**J**) Heart weight to tibia length ratio (HW/TL) measured in vehicle or Torin1 administrated control and mSIRT6-KO mice. *n* = 4–5 mice per group, Data are presented as mean ± s.d, *P < 0.05. (**K**) Fractional shortening measured before and after vehicle or Torin1 administration to control and mSIRT6-KO mice. *n* = 4–5 mice per group, Data are presented as mean ± s.d, *P < 0 05.

To rule out any confounding effects arising due to systemic abnormalities associated with whole-body deletion of SIRT6, we employed a tissue-specific SIRT6-KO mice to study the regulation of mTOR signalling and protein synthesis. We specifically deleted SIRT6 in the myocytes by crossing SIRT6 floxed mice with mice expressing Cre recombinase under the human alpha-skeletal actin promoter (ACTA1-Cre) (Figure 5A). The resultant mSIRT6-KO mice were deficient of SIRT6 only in the skeletal muscle and the heart (Figure 5A). We observed a faint band for SIRT6 in the heart, which is probably from the non-myocyte cells in the heart (Figure 5A). We first tested the protein synthesis levels in these animals by SUnSET assay and observed protein synthesis was significantly higher in the skeletal muscle and the heart of mSIRT6-KO mice compared to the floxed controls (Figure 5B and 5C). Further, in line with the results from the whole body SIRT6 KO mice, we observed increased phosphorylation as well as enhanced protein levels of key components of the mTOR-signalling pathway in the muscle tissues of mSIRT6-KO mice (Figure 5D). Similarly, we observed increased expression of mTOR signalling genes in mSIRT6-KO mice (Figure 5E). These results further confirm the activation of mTOR signalling and protein synthesis under SIRT6 deficient conditions.

We have previously reported that cardiac-specific SIRT6-KO mice develop cardiac hypertrophy in an age-dependent manner (31). Similar to our previous findings, we find that mSIRT6-KO mice develop spontaneous cardiac hypertrophy as evidenced by the increased heart weight to tibia length ratio at 9 months of age (Figure 5F), Further our echocardiographic analysis revealed reduced fractional shortening in these mice, when compared with age-matched controls (Figure 5G), indicating compromised cardiac function. One of the key hallmarks of cardiac hypertrophy is increased protein synthesis, which enables the enlargement of the individual cardiomyocytes (46). Since SIRT6 deficiency leads to enhanced protein synthesis by virtue of increased activation of mTOR signalling, we tested whether pharmacological inhibition of mTOR signalling can rescue cardiac hypertrophy observed under SIRT6 deficient conditions. Our *in vitro* studies on primary cardiomyocytes indicated that treatment with mTOR inhibitor, Rapamycin markedly reduced the expression of hypertrophy marker ANP in SIRT6 depleted cells (Figure 5H and 5I). We then extended the study to *in vivo* by administering mSIRT6-KO mice with mTOR inhibitor, Torin1 for ten days. The heart weight to tibia length ratio was significantly reduced in Torin1 administered mSIRT6-KO mice (Figure 5J), indicating that inhibition of mTOR signalling reduced the cardiac hypertrophy. Further, our results indicate that administration of Torin1 significantly improved cardiac function in mSIRT6-KO mice as evidenced by the improved fractional shortening in these mice (Figure 5K). Collectively these results indicate that hyperactive mTOR signalling contributes to increased protein synthesis and cardiac hypertrophy in SIRT6 deficient mice.

## DISCUSSION

Our work for the first time establishes a novel role for SIRT6 in the transcriptional regulation of mTOR signalling via Sp1 transcription factor. Further, our work demonstrates a novel deacetylase-independent function of SIRT6 in regulating global protein synthesis. These results assume significance in the background of recent studies attributing altered proteostasis to be a major determinant of aging (1,3).

Previous works have demonstrated the role of Sirtuins in cell division, DNA repair, chromatin regulation, transcription, glucose and lipid metabolism (8,47,48). However, the role of Sirtuins in the regulation of protein synthesis is only beginning to be understood (49). We find that overexpression of SIRT6 suppresses protein synthesis in diverse cell types. Surprisingly, SIRT6 attenuates protein synthesis in a manner independent of its catalytic activity. Although most studies find the catalytic activity of SIRT6 to be essential for its functions, few recent reports have demonstrated roles independent of its catalytic activity (50–52). Notably, a recent study reported that overexpression of SIRT6 thwarts the stem-like traits and tumorigenic capacity of human cancer cells independent of the deacetylase activity (50). We believe that future studies in this direction could reveal other interesting deacetylase-independent roles of SIRT6.

Increased protein synthesis is crucial for survival, proliferation, progression and transformation of cancer cells (53). Interestingly, SIRT6 is well-known for its tumour suppressor roles and it has been shown to be downregulated or mutated in multiple cancer types (21,54,55). Consistent with this, SIRT6 deficiency resulted in increased tumour growth, where protein synthesis is highly abnormal (21,54). Further, SIRT6 deficiency is linked to increased ribosome biogenesis in cancer cells driven by the de-repression of the MYC transcription factor (21). Depending on the context, it is possible that SIRT6 might regulate protein synthesis and ribosome biogenesis in a coordinated manner through the transcription factors Sp1 and MYC.

One of the key regulators of protein synthesis is the mTOR signalling pathway (13). Interestingly, we find that SIRT6 deficiency leads to increased levels of total, as well as the phosphorylated forms of the mTOR signalling proteins, contributing to enhanced activation of the pathway. In this scenario, though the phosphorylated to total protein ratio might not be strikingly different between control and SIRT6 deficient conditions, the amount of phosphorylated protein, which correlates with activation is indeed markedly higher in SIRT6 deficient cells. Notably, the phosphorylation of ULK1, which is a direct downstream target of mTOR signalling, was increased upon SIRT6 depletion, while the total levels of ULK1 were not changed. This crucial finding establishes the fact that mTOR signalling is indeed hyperactive under SIRT6 deficient conditions. Further, one of the critical steps involved in the activation of the mTOR signalling involves the translocation of the mTORC1 complex to the surface of lysosomes where the Rheb GTPase binds to and activates the mTOR (56). In line with this, we find that SIRT6 deficiency leads to increased localisation of mTOR and Rheb to the surface of lysosomes. Our findings thus point out that SIRT6 is a critical regulator of the mTOR signalling pathway.

We have previously demonstrated that SIRT6 deficiency in the heart leads to the development of cardiac hypertrophy (31). In the present study, we used this system to establish the link between mTOR signalling and cardiac hypertrophy under SIRT6 deficient conditions. We observe that treatment with Torin1 significantly reduced the heart weight to tibia length ratio and led to improved cardiac function in SIRT6 deficient mice indicating a reversal of hypertrophy. Further, we find that treatment with mTOR inhibitors abrogated the increased protein synthesis observed in SIRT6 depleted cells. Taken together these data establish a critical connection between SIRT6, mTOR signalling, protein synthesis and cardiac hypertrophy.

Our data suggest that CRISPR-mediated inactivation of Sp1 markedly reduces the activity of mTOR signalling, suggesting that Sp1 is critically essential for the regulation of mTOR signalling. However, studies have demonstrated functional overlap between Sp1 and other members of the Sp/KLF transcription factor family, in particular, Sp3 (57). It is possible that other members of this family may also be involved in the regulation of mTOR signalling. Further, mTOR has been shown to be transcriptionally regulated by other transcription factors like Nrf2 (58,59). Given that SIRT6 has been shown to associate with multiple transcription factors (42). we cannot rule out that in addition to Sp1, SIRT6 might co-operate with another transcription factor, to specifically control mTOR signalling. In addition to mTOR signalling genes, our *in-silico* analysis indicates that SIRT6 and Sp1 bind to several common gene promoters across the genome. In fact, we find that the expression of well-known Sp1 targets such as GLUT1 and GLUT3 are upregulated in SIRT6 deficient animals, indicating that SIRT6 might control other Sp1 target genes as well. But one must be cautious in extrapolating these findings to all Sp1 targets since Sp1 associates with many other transcription factors and functions as a part of huge multi-protein complexes which is capable of functioning as transcriptional activators or repressors (44).

Our previous work demonstrated that SIRT6 regulates IGF/Akt signalling genes by deacetylation of H3K9 and c-Jun (31). Contrastingly, in the present study, we do not see any changes in the acetylation status of either Sp1 or H3K9 at the promoters of mTOR signalling genes. Alternatively, we find that SIRT6 influences the occupancy of Sp1 at the mTOR signalling gene promoters by directly binding with the DNA binding domain of Sp1. However, future work is needed to address this interesting phenomenon in regulating these two closely linked signalling pathways by SIRT6 in a different fashion.

In summary, our work demonstrates a novel role for SIRT6 in controlling global cellular protein synthesis via transcriptional regulation of mTOR signalling. We believe that the current model of SIRT6 mediated regulation of protein synthesis will contribute towards understanding and treating diverse pathologies associated with aging.

## Supplementary Material

gkz648_Supplemental_Files
