# Supplementary material for: SIRT6 transcriptionally regulates global protein synthesis through transcription factor Sp1 independent of its deacetylase activity
Source: Nucleic Acids Res. 2019 Aug 2;47(17):9115–31. doi: 10.1093/nar/gkz648 (PMC6755095; doi:10.1093/nar/gkz648)
Supplement: gkz648_Supplemental_Files [file gkz648_supplemental_files.zip › Legends for Supplemental Figures and Files.docx]

**Legends for Supplemental Figures and Files:**

**Supplemental Figure 1:** *A)* Quantitative representation of puromycin incorporation observed in Figure 1*A.* The results are expressed as the fold change relative to controls. n = 4 mice per group. Data are presented as mean ± s.d, * p < 0.05. *B)* Western blotting images depicting depletion of SIRT6 in SIRT6 stable knockdown 293T cells. *C)* Representative images of immunofluorescence SUnSET analysis in null or SIRT6 adenovirus-infected cardiomyocytes in the presence of vehicle or 100 μM phenylephrine (PE) for 24 hrs. Puromycin staining is shown in red, SIRT6 is shown in green and the nuclei stained with Hoechst 33342 are shown in blue. Scale bar = 20 µm. *D)* Representative images of immunofluorescence analysis of puromycin incorporation in control or SIRT6 KD neonatal primary cardiomyocytes treated with vehicle or 100 nM Rapamycin for 24 hrs. Puromycin staining is shown in red, SIRT6 is shown in green and the nuclei stained with Hoechst 33342 is shown in blue. Scale bar = 20 µm. *E and F)* Representative images of immunofluorescence analysis to confirm the SIRT6 depletion (*E*) or overexpression (*F*) in HeLa cells corresponding to the experiment presented in Figure 2*H* and 2*I*. SIRT6 is shown in green and the nuclei stained with Hoechst 33342 is shown in blue. Scale bar = 20 µm.

**Supplemental Figure 2:** *A*) Gene set enrichment analysis of the PID_MTOR_4PATHWAY gene set in the Sp1 ChIP-seq dataset from K562 cells (ENCODE ID - ENCSR991ELG). *B)* Overlapping binding sites of SIRT6 and Sp1 in K562 cell line (SIRT6 - ENCSR000AUB, Sp1 - ENCSR991ELG). *C)* Overlapping binding sites of SIRT6 and Sp1 in hESC line (SIRT6 - ENCSR000AUS, Sp1 - ENCSR000BIR). *D)* SIRT6 and Sp1 ChIP signal in the genomic regions surrounding the transcription start site of mTOR, Rheb, RPS6KB1 (p70S6K) and RPTOR in K562 and hESC cells. The binding was visualized using the Integrated Genome Viewer (IGV). The ENCODE IDs of the datasets are the same as those mentioned in Supplemental figure 2*B* and 2*C*. *E)* Representative images of immunofluorescence analysis to confirm the SIRT6 depletion in HeLa cells corresponding to the experiment presented in Figure 3*K*. SIRT6 is shown in green and the nuclei stained with Hoechst 33342 is shown in blue. Scale bar = 20 µm.

**Supplemental Figure 3:** A) SDS-PAGE showing purified SIRT6 and Sp1 ZFDBD used in NMR titration experiments. B) Overlay of 2D ^15^ N- ^1^ H TROSY-HSQC NMR spectra of free Sp1 ZFDBD (black) with Sp1 ZFDBD in complex with SIRT6 (in red) (at 1:2 molar ratio). The assignment of the peaks residues that did not disappear upon addition of SIRT6 is shown. Majority of these residues belong to zinc finger 3 of Sp1 ZFDBD. The assignment is taken from a previously published study (Oka S. et. al. 2004). C) SDS-PAGE showing purified SIRT6 H133Y mutant used in NMR titration experiments

**Supplemental File 1:** Overlaps of different transcription factor targets (TFT) gene sets with the PID_MTOR_4PATHWAY gene set.

**Supplemental File 2:** Bioinformatic analysis for the presence of evolutionarily conserved Sp1 binding sites in the promoter of mTOR signalling genes.

**Supplemental File 3:** Details of primers, sequences and other key resources/reagents used in the study.
