# Supplementary material for: SIRT6 transcriptionally regulates global protein synthesis through transcription factor Sp1 independent of its deacetylase activity
Source: Nucleic Acids Res. 2019 Aug 2;47(17):9115–31. doi: 10.1093/nar/gkz648 (PMC6755095; doi:10.1093/nar/gkz648)
Supplement: gkz648_Supplemental_Files [file gkz648_supplemental_files.zip › Supplemental File 2.pdf]

Supplemental File 2 - Evolutionarily conserved Sp1 binding sites in the promoter of mTOR signaling genes

RHEB

```
Human           TAGCGTTTCGCCAATCACCGCACCTCCCACCTAT-TGCCCCGCCCTGCGCT--CCGTTCT
Rhesus Monkey   CAGCTTATCACCAATCACCGCACCTCCCACCTAT-TGCCCCGCCCTGCGCT--CCGTTCC
Chimp           TAGCGTTTCGCCAATCACCGCACCTCCCACCTAT-TGCCCCGCCCTGCGCT--CCGTTCT
Cow             CAGAGGCCCACCAATCACCGCACCTCCCACGCGT-CTCCCCGCCCCCTCCCC--GTGATC
Pig             CAGCGTTCTACCAATCACCTCACCTCTCAA-TCT-TTCCCCGCCCCTTCCC--CCGATCT
Dog             CAACCTCCCGCCAATCACTGCGCTCCCCACAACC-TGCCCCGCCCC-CGCC--CTGCCCT
Mouse           CAGCACTTCACCAATCACCGTGCTTCACGCCTCT-GGTTCCGCCCCCGCAC--CCACATT
Rat             CGTCACTTCACCAATGACCGCGCTTCTCGCCTAT-GGTTCCGCCCC-CGCACGCCATATC
```

mTOR

```
Human           ACTCACGACCGATTGGTTCTCCCGAGTGGA /40/ GTCTATTTGAACAGTCCCCGCCCCCTGGAG
Rhesus Monkey   ACTCACGACCGATTGGTTCTCCCGAGTGGA /40/ GTCTATTTGAATAGTCCCCGCCCCCTGGAG
Chimp           ACTCACGACCGATTGGTTCTCCCGAGTGGA /40/ GTCTATTTGAACAGTCCCCGCCCCCTGGAG
Cow             ACTCACGAATGATTGGTTCTCCCGAGAGGA /40/ GTCTATTTGAACAGTCCCCACCCCCTGGAG
Pig             ACTCACCAGCGATTGGTTTTTTCGAGAGGA /40/ GTCTATTTGAATAGTCTCCGCCCCGCGGAG
Dog             GCTCGCGACCCATTGGCTCCCCCGAATGGA /40/ GTCTGTTTGAACAGTCCCCGCCCCCCGGAG
Mouse           ACTCACGACTGATTGGCTCTTCGGAATAGT /40/ GTCTATTTGAACAATCCCCGCCCTCGAGAA
Rat             ACTCACGACTGATTGGCTCTCCGGAATACT /40/ GTCTATTTGAACAATCCCCGCCCCC--GAG
Rabbit          ACTCGCGCGCGATTGGTTCTCCCGAGCGAA /40/ GTCTATTTGAACAGTCCCCGCCCTCTGGAG
Horse           ACTCACGACCGATTGGCTCTCCCGAGCGAA /40/ GTCTATTTGAACAGTCCCCGCCCCCTGG-G
Opposum         ACTCGCCGCTGATTGGTCTATCCAGGTGTT /40/ GTCCATTTGAACTTATCCCGCCCCTCTGAG
```

P70S6K

```
Human           CGGGTCCGGGCCCATGAGGCGACGAAGGAGGCGGGACGGCTTTTACCCAGCCCCGGACTT
Rhesus monkey   CGGGTCCGGGCCCATGAGGCGACGAAGGAGGCGGGACGGCTTTTACCCAGCGCCGGACTT
Mouse           CGGGTCCGGGCCCATGAGGCGACGACGGAGGCGGGACGGCTTTTACCTAGCGCCTGACTT
Rat             CGGGTCCGGGCCCATGAGGCGACGACGGAGGCGGGACGGCTTTTACCCAGCGCCTGACTT
Horse           CGGGTCCGGGCCCATGAGGCGACGACGGAGGCGGGACGGCTTTTACCCAGCGCCGGACTT
Cow             CGGGTCCGGGCCCATGAGGCGACGACGGAGGCGGGACGGTTTCTACCCAGCGCCGGACTT
Pig             CGGGTCCGGGCCCATGAGGCGACGACGGAGGCGGGACGGCTTTT----------------
Dog             CGGGTCCGGGCCCATGAGGCGACGACGGAGGCGGGAC-----------------------
Opossum         CGGGTCCGGGCCCATGAGGCGGCGGCGGCGGCGGGACGGATTTTATCCAGCGCCAGACTT
```

Supplemental file 2 - Sp1 binding sites are shown in red and CAAT box signals which enhance transcription are shown in blue. Numbers within slashes indicate the number of bases in between the sequences that have been skipped in the representation.
