# Supplementary material for: SIRT6 transcriptionally regulates global protein synthesis through transcription factor Sp1 independent of its deacetylase activity
Source: Nucleic Acids Res. 2019 Aug 2;47(17):9115–31. doi: 10.1093/nar/gkz648 (PMC6755095; doi:10.1093/nar/gkz648)
Supplement: gkz648_Supplemental_Files [file gkz648_supplemental_files.zip › Supplemental File 3 - Key resources table.pdf]

Supplemental File 3: Details of primers, sequences and other key resources

Mouse q-PCR primers

mTOR For	CACAAGGAGATCCGCATGGA
mTOR Rev	GCGGATATCAGGGTCAGGAT
Rheb For	GGTCTGTGGGAAAGTCCTC
Rheb Rev	TGTTCTCTATGGTTGGATCGT
p70S6K For	CACCTGTCAGCCCAGTCAAA
p70S6K Rev	CCGCTCACTGTCACATCCAT
RAGA For	GAACCTGGTGCTGAACCTGT
RAGA Rev	GATGGCTTCCAGACACGATT
RAGB For	TTCGATTTCTGGGAAACCTG
RAGB Rev	AGTTCACGGCTCTCCACATC
RAGC For	AGATGTCACCCAATGAGACTCT
RAGC Rev	AGTCGTCCTGTGCATCAATGA
RAGD For	AGGAGCGGCAAGTCGTCTAT
RAGD Rev	CCGGCAGATCCTGTTGGTG
RAPTOR For	CTTCCAACAACACGAGGAGC
RAPTOR Rev	TCTCCCGGTCAGTATCCCAG
TSC1 For	CAGGAGTTACAGACAAAGCTGG
TSC1 Rev	AGCTTCTGAGAGACCTGGCT
TSC2 For	CCGCAACCTGTCCTTTGTGG
TSC2 Rev	AATGTGGCGGAGTCTTGCGA
Akt1 For	TGGACAAGGACGGGCACATCAAG
Akt1 Rev	TACTCCGGCGTTCCGCAGAATG
Actin For	CACTGTCGAGTCGCGTCC
Actin Rev	TCATCCATGGCGAACTGGTG
GAPDH For	TGCAGTGGCAAAGTGGAGATT
GAPDH Rev	TTGAATTTGCCGTGAGTGGA

Other q-PCR primers

Renilla Luciferase For	CGGATGATAACTGGTCCGCA
Renilla Luciferase Rev	TAATACACCGCGCTACTGGC
Human GAPDH For	TCAAGATCATCAGCAATGCC
Human GAPDH Rev	CGATACCAAAGTTGTCATGGA

Human ChIP primers

mTOR For	CCGATTGGTTCTCCCGAGTG
mTOR Rev	GAAGAGAAGCTTCAGGACCCG
Rheb For	GAACCCGCCAACCGCTTA
Rheb Rev	GGTGATTGGCCAGAACGGA
RPTOR For	GACCGCTCTGAGGAGGTTTG
RPTOR Rev	CCATCAAGGAGGACGCCATA
p70S6K For	CCTAAGCAGCCGGTGATGG
p70S6K Rev	CTCCTTCGTCGCCTCATGG
RAGA For	TTCCATCTTAGGGCTCACGC
RAGA Rev	GGAGATACGCTCGCTTTCCG
RAGB For	TGACAGACTCCGGGGTAAGG
RAGB Rev	TAGGCACTAAGCGGAGAACG
RAGC For	ACGTGACGGGGCACCA
RAGC Rev	AAGCCCAGCCGCTTATTGG
RAGD For	TGAGTCTGGGAGGGTGCAG
RAGD Rev	CGAGTCACCTGACACACACT
TSC2 For	CTCCTGCGGACTACACATCC
TSC2 Rev	AGTTGTAGTTCTGTGCCGCC

SIRT6 site directed mutagenesis primers

SIRT6-H133Y For	CTTCCACAAACATGTTCCCATAGAGCTCTGCCAGTTTGTCC
SIRT6-H133Y Rev	GGACAAACTGGCAGAGCTCTATGGGAACATGTTTGTGGAAG
SIRT6-S56Y For	GGATGCCAGAGGCAGTATAGATGCCGGCACCCGTG
SIRT6-S56Y Rev	CACGGGTGCCGGCATCTATACTGCCTCTGGCATCC

siRNA sequences

Human SIRT6 siRNA	AAGAAUGUGCCAAGUGUAAGA
Rat SIRT6 siRNA	GCAUCUCAAUGGUUCCUAU
Control siRNA	AAUUCUCCGAACGUGUCACGU

Key resources used in the study

Designation	Reagent / Resource type	Source	Catalog number/ Identifier
Sirt6 knockout mice	Mice	Jackson Laboratories, USA,	129-Sirt6tm1Fwa/J
SIRT6 flox/flox mice	Mice	Jackson Laboratories, USA	Sirt6tm1.1Cxd/J
ACTA-Cre mice	Mice	Jackson Laboratories, USA	FVB.Cg-Tg(ACTA1-cre)79Jme/J
anti-Rheb	Antibody	Abcam	25873
anti-phospho mTOR	Antibody	Cell Signaling Technology	5536
anti-mTOR	Antibody	Cell Signaling Technology	2983
anti-phospho TSC2	Antibody	Cell Signaling Technology	3617
anti-TSC2	Antibody	Cell Signaling Technology	4308
anti-phospho p70S6K	Antibody	Cell Signaling Technology	9205
anti- p70S6K	Antibody	Cell Signaling Technology	2708
anti-phospho 4EBP1	Antibody	Cell Signaling Technology	2855
anti-4EBP1	Antibody	Cell Signaling Technology	9644
anti-SIRT6	Antibody	Cell Signaling Technology	12486
anti-SP1	Antibody	Cell Signaling Technology	9389
anti-Puromycin	Antibody	Developmental Studies Hybridoma Bank	PMY-2A4
anti-LAMP2	Antibody	Developmental Studies Hybridoma Bank	H4B4
anti-β -actin (HRP-conjugate)	Antibody	Sigma-Aldrich	A3854
anti-GAPDH	Antibody	Santa Cruz Biotechnology	sc-25778
anti-Acetylated-Lysine	Antibody	Cell Signaling Technology	9441
anti-rabbit HRP	Antibody	Santa Cruz Biotechnology	sc-2004
anti-mouse HRP	Antibody	Santa Cruz Biotechnology	sc-2005
anti-rabbit HRP	Antibody	Cell Signaling Technology	7074
anti-mouse HRP	Antibody	Cell Signaling Technology	7076
Normal Rabbit IgG	Antibody	Cell Signaling Technology	2729

Donkey anti-mouse, Alexa Fluor 488	Antibody	Thermo Fisher Scientific	A-21202
Goat anti-rabbit, Alexa Fluor 546	Antibody	Thermo Fisher Scientific	A-11035
Clean-Blot IP Detection Reagent	Antibody	Thermo Fisher Scientific	21230
pcDNA3.1	Plasmid	Invitrogen	V790-20
SIRT6 Flag	Plasmid	Addgene	13817
pHAGE-CMV-Rheb(D60K)-IRES-eGFP-W	Plasmid	Addgene	32522
pN3-Control	Plasmid	Gifted by Dr. Guntram Suske, IMT, Marburg	
pN3-Sp1FL	Plasmid	Gifted by Dr. Guntram Suske, IMT, Marburg	
pGL3-promoter	Plasmid	Gifted by Dr. Guntram Suske, IMT, Marburg	
pRL-CMV	Plasmid	Promega	E2261
pAmpho	Plasmid	Clontech	
pSUPERretro-Sirt6 shRNA1	Plasmid	Addgene	53147
pSuperRetro control	Plasmid	Addgene	11189
Ad-Null	Adenovirus	Vector Biolab	1300
Ad-SIRT6 (human)	Adenovirus	Vector Biolab, Adenovirus	1556
Qubit dsDNA HS assay kit	commercial assay or kit	Thermo Fisher Scientific	Q32851
Dual-Luciferase® Reporter Assay System	commercial assay or kit	Promega	E1910
SimpleChIP® Enzymatic Chromatin IP Kit	commercial assay or kit	Cell Signaling Technology	9003
cDNA Synthesis Kit	commercial assay or kit	Thermo Fisher Scientific	K1621
QuikChange Site-Directed Mutagenesis Kit	commercial assay or kit	Agilent Technologies	200518
nProtein A Sepharose® 4 Fast Flow	Chemical / Reagent	GE Healthcare	17-5280-01
ANTI-FLAG® M2 Affinity Gel	Chemical / Reagent	Sigma-Aldrich	A2220
Trizol	Chemical / Reagent	Sigma-Aldrich	T9424
SYBR® Premix Ex Taq™	Chemical / Reagent	TaKara	RR420L
cOmplete™, Mini Protease Inhibitor Cocktail	Chemical / Reagent	Sigma-Aldrich, ROCHE	11836153001
(R)-(-)-Phenylephrine hydrochloride	Chemical / Reagent	Sigma-Aldrich	P6126

(-)-Isoproterenol hydrochloride	Chemical / Reagent	Sigma-Aldrich	I6504
Rapamycin	Chemical / Reagent	Cayman Chemical	13346
Torin1	Chemical / Reagent	Cayman Chemical	10997
MithramycinA	Chemical / Reagent	Cayman Chemical	11434
Puromycin	Chemical / Reagent	VWR	J593
Cycloheximide	Chemical / Reagent	Amresco	94271
IGF-1	Chemical / Reagent	Thermo fisher Scientific	PHG0071
[^{3}H]-Leucine	Chemical / Reagent	Amersham Biosciences	TRK510
^{35}S-Invivo Pro Twinlabel mix	Chemical / Reagent	Bhabha Atomic Research Centre, India	LCS8
Dulbecco's Modified Eagle's Medium- High glucose	Cell culture media	Sigma-Aldrich	D5648
Leucine-free DMEM	Cell culture media	Thermo fisher Scientific	30030
Methionine-free DMEM	Cell culture media	Sigma-Aldrich	D0422
Lipofectamine™ 2000 Transfection Reagent	Chemical / Reagent	Thermo Fisher Scientific	11668019
Lipofectamine™ RNAiMAX Transfection Reagent	Chemical / Reagent	Thermo Fisher Scientific	13778150
Horse serum, heat inactivated	Cell culture reagent	Thermo Fisher Scientific	26050088
Fetal Bovine Serum	Cell culture reagent	Thermo Fisher Scientific	10500064
Antibiotic-Antimycotic mixture	Cell culture reagent	Thermo Fisher Scientific	15240062
ProLong Gold Antifade Mountant with DAPI	Chemical / Reagent	Thermo Fisher Scientific	P36931
Hoechst 33342	Chemical / Reagent	Thermo Fisher Scientific	H3570
Clarity ECL Western Blotting Substrate	Chemical / Reagent	BioRad	5060
Amersham Hybond PVDF membrane	Miscellaneous	GE Healthcare	10600023
Visual Sonics high-frequency ultrasound system	Equipment	Vevo 1100	
ChemiDoc™ Touch Imaging System	Equipment	BioRad	
ImageQuant™ LAS 4000	Equipment	GE Healthcare	

QuantStudio™ 6 Flex Real-Time PCR System	Equipment	Thermo fisher Scientific	
CFX384 Touch™ Real-Time PCR Detection System	Equipment	BioRad	
LSM 880 confocal microscope	Equipment	Zeiss	
BioComp gradient master	Equipment	BioComp Instruments	
SW41 rotor and Beckman LS-80M Ultracentrifuge	Equipment	Beckman	
Luminometer	Equipment	Turner Machine, TD-20/20	
Typhooon FLA9500 Phosphorimager	Equipment	GE Healthcare	
