# Supplementary figures and images for: SIRT6 transcriptionally regulates global protein synthesis through transcription factor Sp1 independent of its deacetylase activity
Source: Nucleic Acids Res. 2019 Aug 2;47(17):9115–31. doi: 10.1093/nar/gkz648 (PMC6755095; doi:10.1093/nar/gkz648)

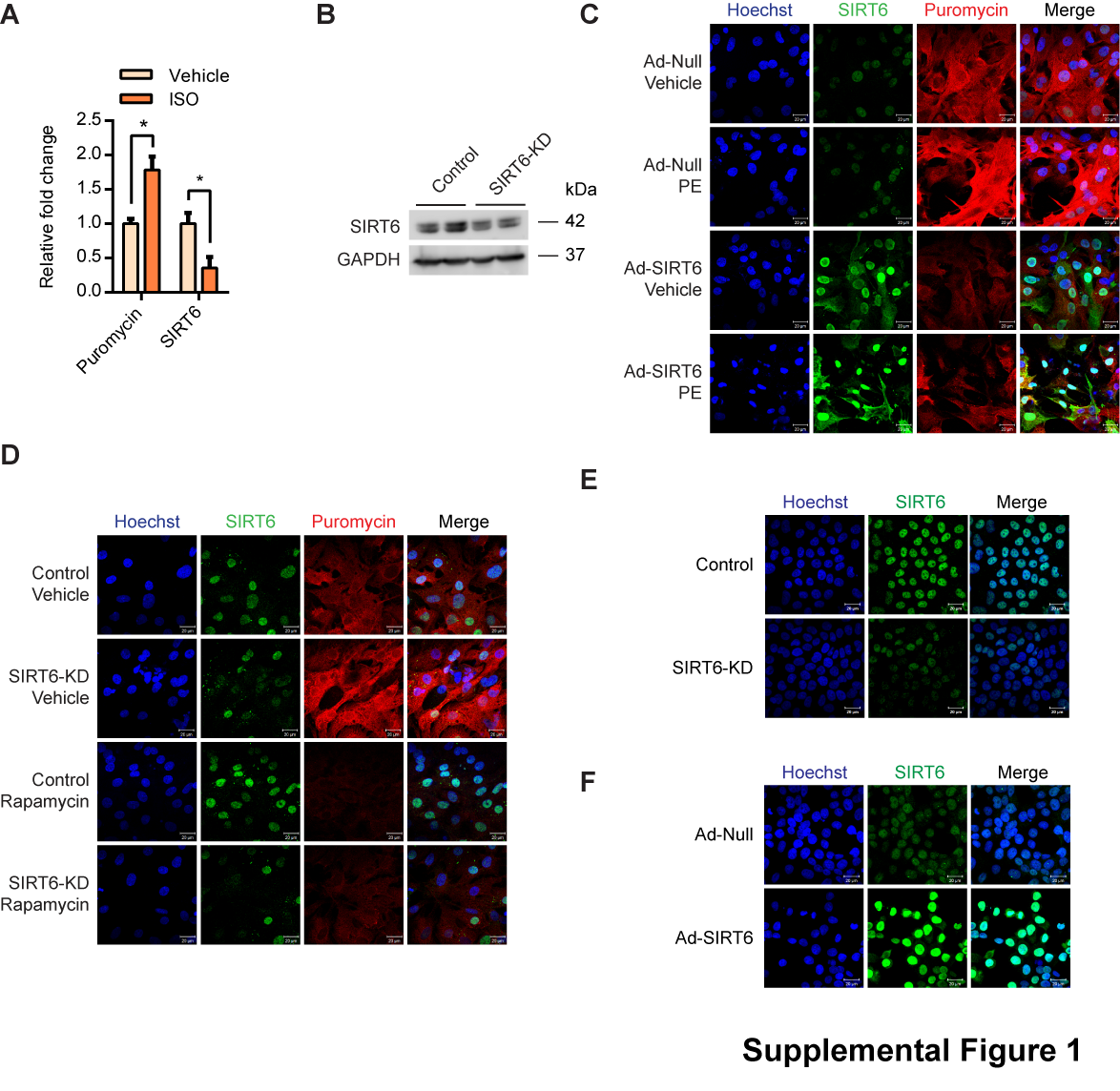

Supplement: gkz648_Supplemental_Files [file gkz648_supplemental_files.zip › Supplemental Figure 1.tif]

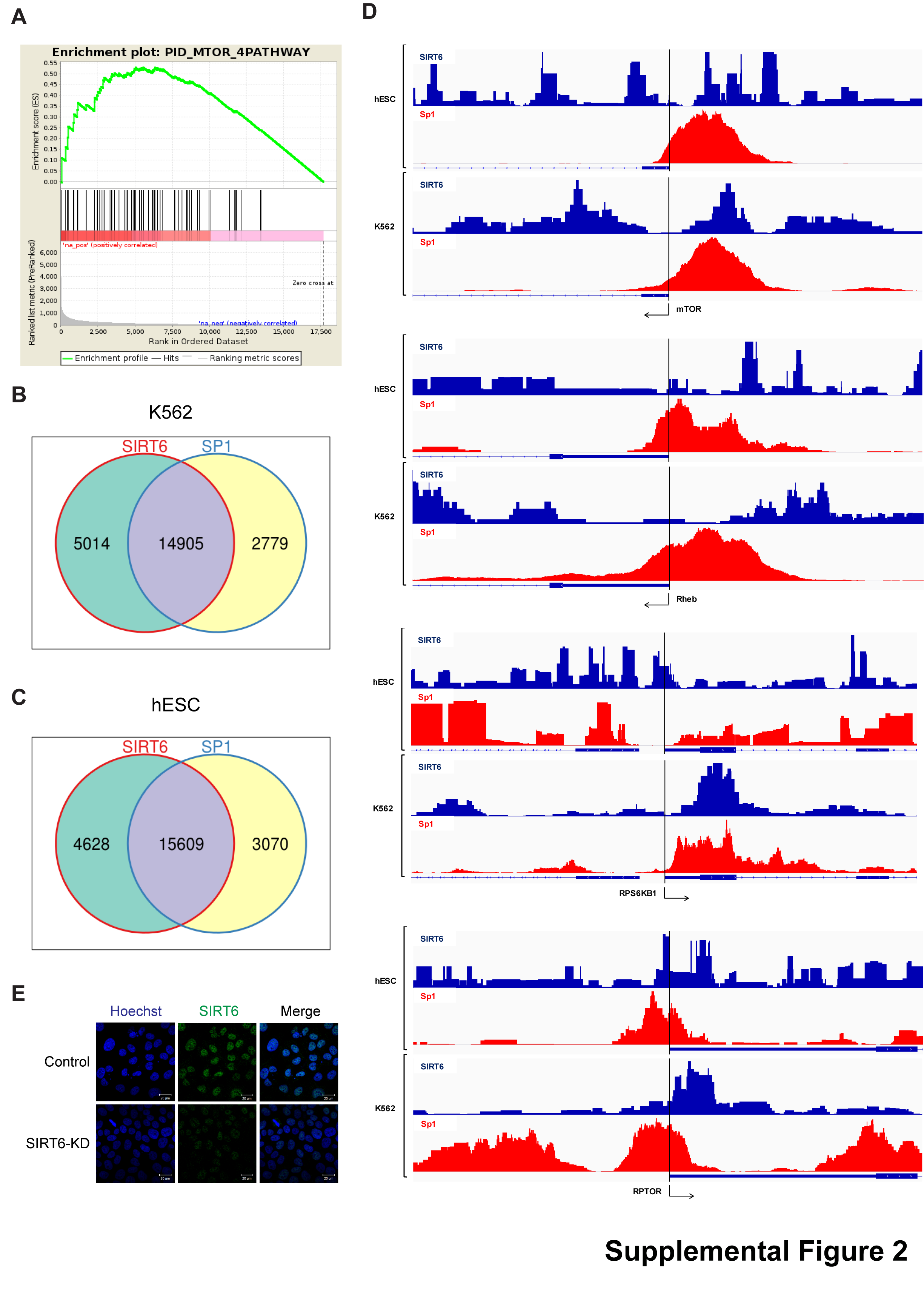

Supplement: gkz648_Supplemental_Files [file gkz648_supplemental_files.zip › Supplemental Figure 2.tif]

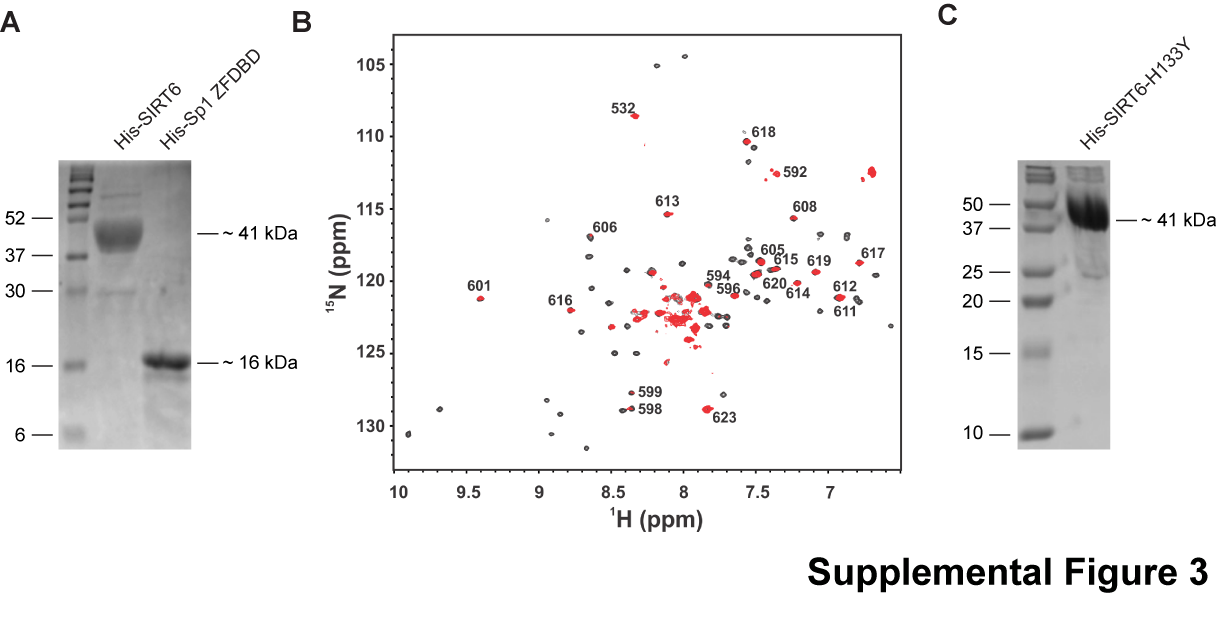

Supplement: gkz648_Supplemental_Files [file gkz648_supplemental_files.zip › Supplemental Figure 3.tif]
